# Attempted characterisation of phenanthrene-4,5-quinone and electrochemical synthesis of violanthrone-16,17-quinone. How does the stability of bay quinones correlate with structural and electronic parameters?†

**DOI:** 10.1039/d0ra06519f

**Published:** 2020-10-15

**Authors:** Dylan Wilkinson, Giacomo Cioncoloni, Mark D. Symes, Götz Bucher

**Affiliations:** School of Chemistry, University of Glasgow, University Avenue Glasgow G12 8QQ UK goetz.bucher@glasgow.ac.uk

## Abstract

In bay quinones, two carbonyl moieties are forced into close proximity by their spatial arrangement, resulting in an interesting axially chiral and nonplanar structure. Two representatives of this little-explored class of compounds were investigated experimentally in this work. Electrochemical oxidation of 4,5-dihydroxyphenanthrene failed to provide evidence for the reversible formation of phenanthrene-4,5-quinone. Even at temperatures as low as *T* = 229 K, cyclic voltammograms did not show any evidence for reversibility, indicating that phenanthrene-4,5-quinone likely is a reactive intermediate even at low temperatures. Electrochemical oxidation of the larger homologue 16,17-dihydroxyviolanthrone, on the other hand, was reversible, and the quinone could be characterised by spectroelectrochemical means. The results of quantum chemical calculations confirm the experimental findings and indicate that a bay dicarbonyl moiety, also found in a number of angucycline antibiotics, does not necessarily have to confer extreme reactivity. However, in a series of phenanthrene quinones with an equal number (zero) of Clar sextets and a varying number of bay carbonyl groups (zero to two), there was a clear correlation between the triplet energy, taken as a measure of biradical character, and the number of bay carbonyl moieties, with the lowest triplet energy predicted for phenanthrene-4,5-quinone (two bay carbonyl moieties).

## Abstract 2

Bay quinones have the carbonyl oxygen atoms in the bay region of polycyclic aromatic hydrocarbons. This study presents cyclic voltammetry and computational work helping to understand when bay quinones can be expected to be isolable, and when not.

## Graphical abstract

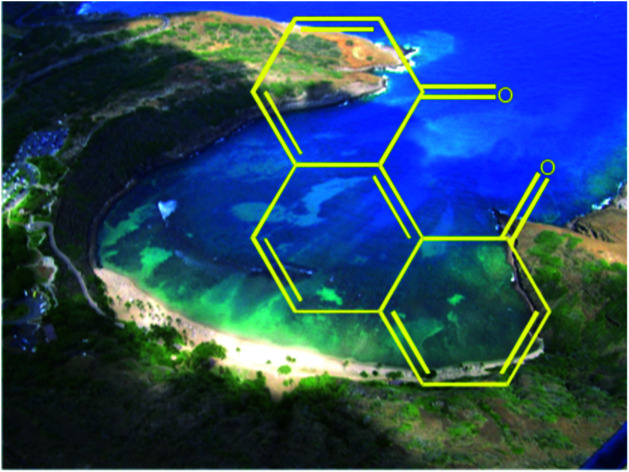

## Introduction

Quinones are a class of mildly oxidising compounds, characterized by the presence of a six-membered ring or a sequence of annelated six-membered rings, with two oxygen atoms bound as carbonyl groups. The simplest quinones are *p*-benzoquinone 1 and *o*-benzoquinone 2, where the *p*-quinoid system in 1 is far more stable than the *o*-quinoid system in 2. The reactivity of quinones, *e.g.*, in their practical use as dehydrogenating agents, is explained in terms of biradicaloid resonance structures such as 1b or 2b. Over the years, many quinones have been synthesized, and this mature field of research has been reviewed authoritatively.^1–4^
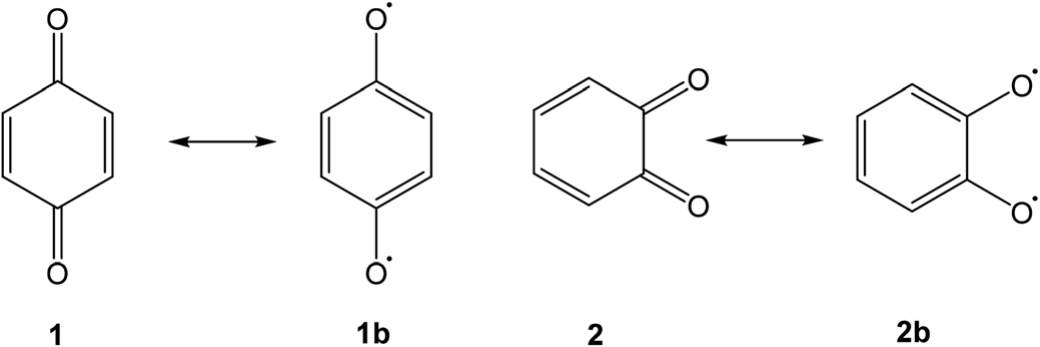


**Scheme 1:**
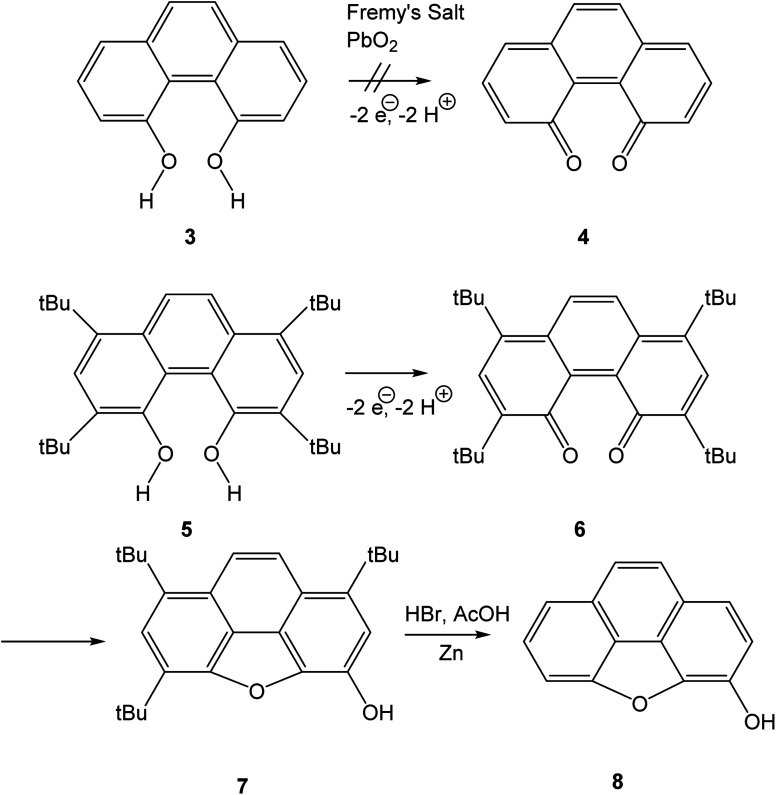
Published attempts at synthesising phenanthrene-4,5-quinone 4 and derivatives.

Bay quinones are a little explored sub-class of quinones. The close spatial interaction of the two oxygen atoms forces the system out of planarity, resulting in reduced π-overlap and strain. The synthesis of the simplest bay quinone, phenanthrene-4,5-quinone 4, had been attempted several times, but without success. Oxidation of 4,5-dihydroxyphenanthrene 3 with a range of oxidants, such as PbO_2_ or Fremy's salt, merely yielded either a derivative of phenanthrene-1,4-quinone or intractable tars (Scheme 1).^5^ Adding methyl groups to the dihydroxyphenanthrene did not improve matters.^6^ Somewhat more successful was the attempted synthesis of a *t*-butylated derivative 6, where oxidation of the dihydroxyphenanthrene precursor 5 instead yielded the rearranged product 7. The latter could then be de-*t*-butylated to yield the natural product morphenol 8. During the oxidation of 5, the authors noted the appearance of a green intermediate, but could not characterise this intermediate by NMR spectroscopy.^7^

**Scheme 2:**
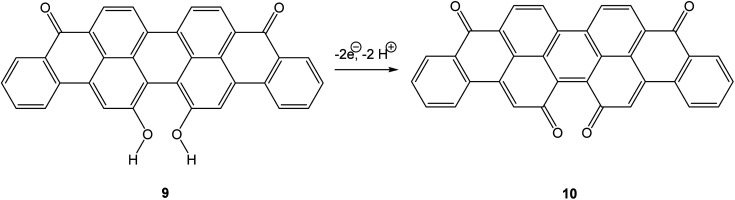
Oxidation of 16,17-dihydroxyviolanthrone 9.

The bay quinone or -dicarbonyl substructure is not limited to reactive species such as phenanthrene-4,5-dione. Among angucycline^8^ antibiotics, there are several examples of compounds showing two carbonyl groups in a bay arrangement, such as tetrangomycin^9^ or oviedomycin.^10^ A bay quinone subunit has also been reported to be formed upon photooxidation of alkynyl-perylenes.^11^ The current work intends to shed light on the reactivity of bay quinones, attempting the characterisation of parent phenanthrene-4,5-quinone 4 by spectroelectrochemical measurements at low temperature. We will also investigate the larger system 16,17-dihydroxyviolanthrone 9/violanthrone-16,17-quinone 10*via* cyclic voltammetry and spectroelectrochemical measurements. Bay quinone 10 is an intermediate in the industrial synthesis of vat dye 9,^12–14^ and therefore its electrochemistry is of more than academic interest (Scheme 2).

In addition to the experimental work, we also present results of DFT calculations on a series of bay quinones, shedding light on structure–reactivity relationships in this class of compounds.

## Results and discussion

### Electrochemistry

**Fig. 1:**
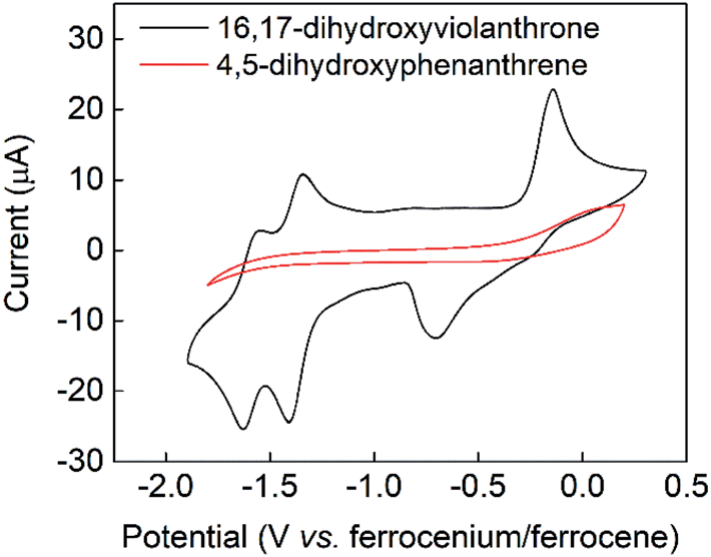
Cyclic voltammetry in dimethylformamide/0.1 M TBAPF_6_ at room temperature and a scan-rate of 100 mV s^−1^. See Experimental section for further experimental details. Black trace: 3 mM 16,17-dihydroxyviolanthrone, red trace: 15 mM 4,5-dihydroxyphenanthrene.

The cyclic voltammogram of 16,17-dihydroxyviolanthrone 9 in dry dimethylformamide (containing 100 mM TBAPF_6_) is shown in Fig. 1 (black trace). There are two fairly reversible reduction waves (peak separation ∼70 mV) at −1.4 V and −1.6 V (*vs.* ferrocenium/ferrocene) possibly corresponding to the reduction of the two carbonyl moieties. There also is a quasi-reversible oxidation event whose mid-point lies near −0.4 V, but whose oxidative and reductive processes are rather widely separated (by about 550 mV).

A scan-rate dependency study on this quasi-reversible wave over the range 10–1000 mV s^−1^ suggests that the broad reduction peak may in fact contain two waves whose relative ratio changes with scan rate (Fig. S1, see ESI†). This is borne out by a cyclic voltammogram at low temperature (−44 °C), which clearly shows that this reductive feature has two peaks (see Fig. S2, ESI†). However, even at scan rates as high as 1000 mV s^−1^, this wave remains only electrochemically quasi-reversible at room temperature, with very little reduction in the peak-to-peak separation. Hence it seems that the electron transfer kinetics for this process are slow, with some evidence for rapid non-electrochemical re-organisation process(es) occurring after the oxidation event.

**Fig. 2:**
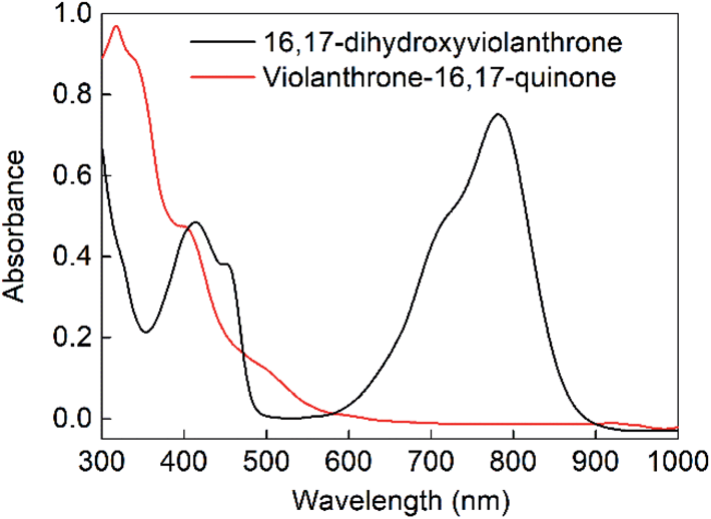
Comparison between the electronic spectra of fresh 16,17-dihydroxyviolanthrone (black) and the same sample after electrochemical 2-electron oxidation to violanthrone-16,17-quinone (red). The samples were diluted to approximately 20 μM in order to collect these spectra.

Bulk electrolysis was conducted on the oxidation event occurring at −0.15 V by applying a potential of 0 V (*vs.* ferrocenium/ferrocene) as shown in Fig. S3a (ESI†). This showed that the oxidative wave was a two-electron process, with the bulk electrolysis consuming around 90% of the charge expected on the basis of an oxidation by 2 electrons per molecule. This oxidation was accompanied by a colour change from dark green to dark yellow (see Fig. 2). Upon reduction of this oxidised sample at −1 V, charge consistent with a two-electron reduction was passed (see Fig. S3b, ESI†) and the original green colour was restored. Hence the electrochemically quasi-reversible wave centred at around −0.4 V appears to be a two-electron process whereby reduction of the oxidised species restores the original molecule. This supposition is supported by UV-Vis analysis of the original, oxidised and re-reduced forms (as shown in Fig. 2 and S4†), which confirms that all the salient features of the fresh 16,17-dihydroxyviolanthrone are restored upon re-reduction.

In contrast to the fairly well-defined behaviour of 16,17-dihydroxyviolanthrone, the simpler analogue 4,5-dihydroxyphenanthrene 3 does not show any similar reversible electrochemistry over the potential range shown in Fig. 1 (red trace), even at a low temperature of *T* = −44 °C. Indeed, on this basis, there is no evidence even for the formation of phenanthrene-4,5-quinone 4, let alone its re-reduction to 4,5-dihydroxyphenanthrene 3.

### Results of calculations

**Fig. 3:**
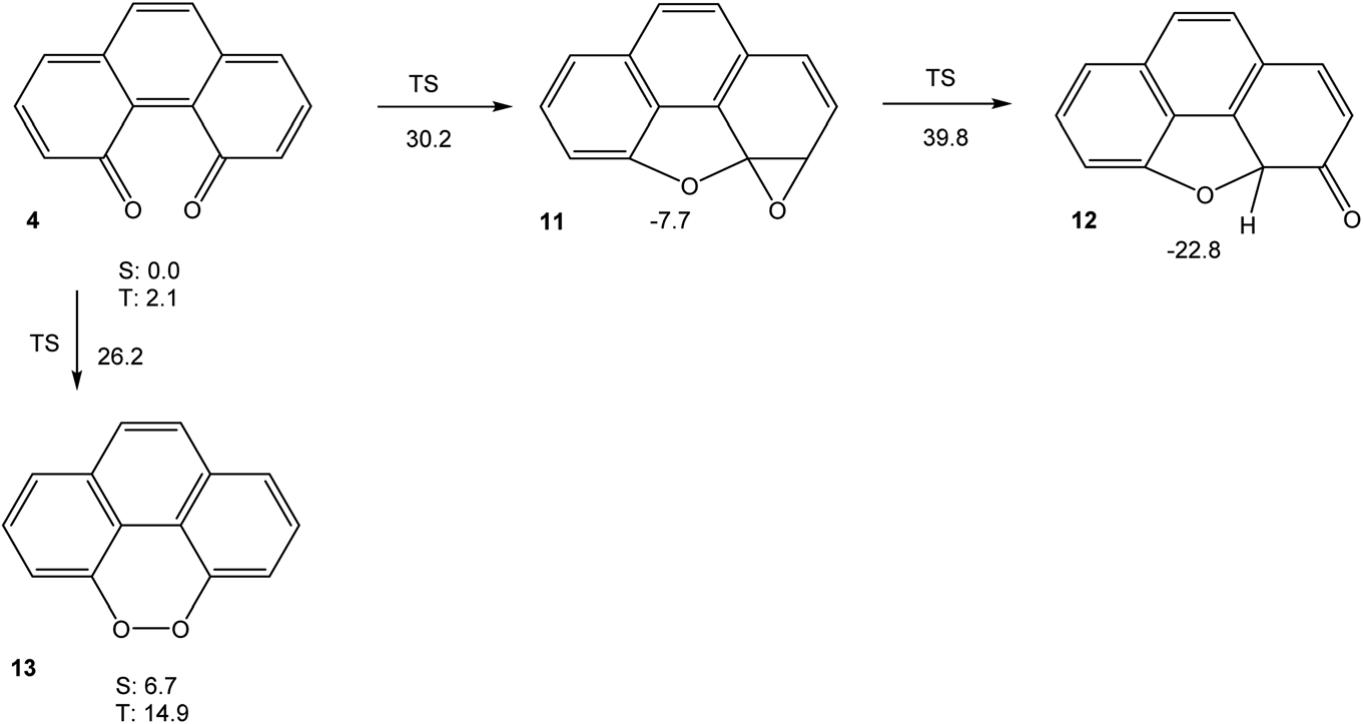
Free energies (in kcal mol^−1^) of relevant stationary points on the potential energy hypersurface of 4 (M06-2X/cc-pVTZ). All stationary points are on the singlet spin manifold, unless indicated otherwise.

In order to understand the very high reactivity and short-lived nature of phenanthrene-4,5-quinone 4, we used density functional theory (M06-2X/cc-pVTZ) to explore its potential energy hypersurface. The results are shown in Fig. 3.

The results shown in Fig. 3 indicate that the intramolecular cyclization of 4, yielding ketone 12 (which would then tautomerise to 8) *via* oxirane 11, would suffer from significant barriers. The calculated triplet free energy of 4, on the other hand, is extremely small with Δ*G*_T_ = 2.1 kcal mol^−1^. This value indicates that the quinone should have significant biradical-type reactivity. Thus, our experimental failure in detecting 4*via* electrochemical means, even at low temperatures, does not come as surprise. If the intermolecular reactivity of phenanthrene-4,5-quinone is impeded by steric protection, as in 6, however, the sequence of cyclization reactions shown in Scheme 1 should be feasible, in agreement with the findings by Hewgill and Stewart (see Scheme 1).^7^ A calculation of the UV-Vis spectrum of 4 (TD-B3LYP/6-311++G(d,p)//M06-2X/cc-pVTZ) gives a longest-wavelength absorption maximum of *λ* = 696 nm, corresponding to a deep green colour (for the full spectrum, see Fig. S5, ESI†). Hence, the temporary observation of a green colour during the oxidation of 5 can reasonably be attributed to the formation of 6, while their failure to detect the species by NMR spectroscopy probably is due to the lowest triplet excited state of 6 being so low in energy that it is thermally populated, which would significantly broaden its NMR bands.^7^ As a final note, the peroxide 13, which is a valence tautomer of 4, is predicted to be a minimum structure, albeit higher in energy than 4. Unlike the severely twisted singlet ground and first triplet excited state of quinone 4, the first triplet excited state of 13 is calculated to be planar (point group *C*_2v_). The barrier for interconversion of 4 and 13 on the singlet spin manifold is significant. On the triplet spin manifold, we were unable to locate a transition state, presumably because the symmetry-breaking reaction ^3^13 → ^3^4 has to go *via* a valley-ridge inflection point.

**Fig. 4:**
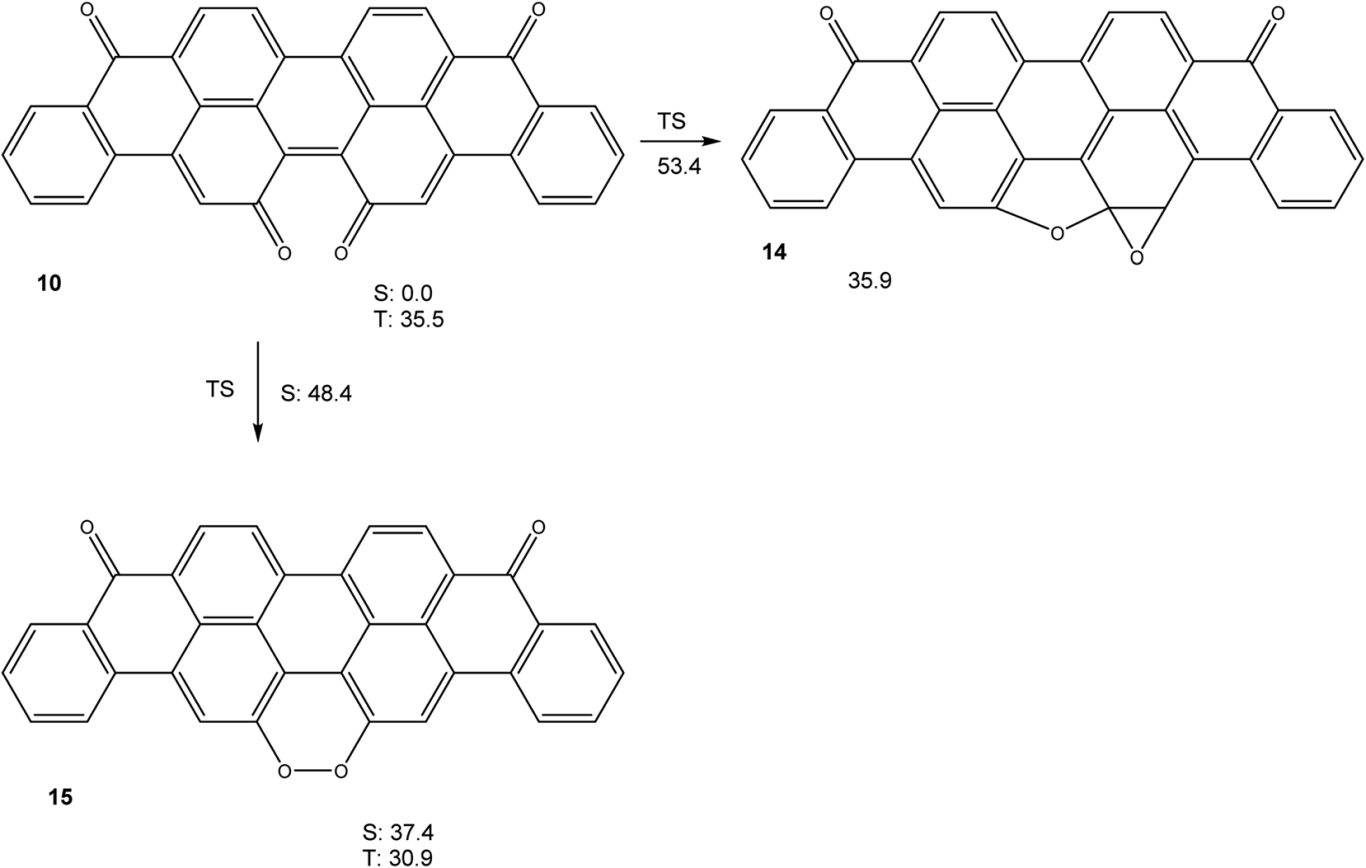
Free energies (in kcal mol^−1^) of relevant stationary points on the potential energy hypersurface of 10 (M06-2X/cc-pVTZ). All stationary points are on the singlet spin manifold, unless indicated otherwise.

Calculations (M06-2X/cc-pVTZ) on the potential energy hypersurface of violanthrone-16,17-quinone 10 gave the results shown in Fig. 4.

The results shown in Fig. 4 show that a cyclization sequence 10 → 14 should not be viable in this system, as it is both endothermic and inhibited by a very large barrier. Unlike quinone 4, 10 does not have an unusually small triplet free energy, as a value of Δ*G*_T_ = 35.5 kcal mol^−1^ is rather typical of large annelated aromatic hydrocarbons. Like in the system 4/13, a valence tautomerism exists with peroxide 15. Remarkably, this peroxide is predicted to have a triplet ground state, with the first singlet excited state being higher in free energy by Δ*G*_S_ = 6.5 kcal mol^−1^, in spite of being of closed shell nature.

**Fig. 5:**
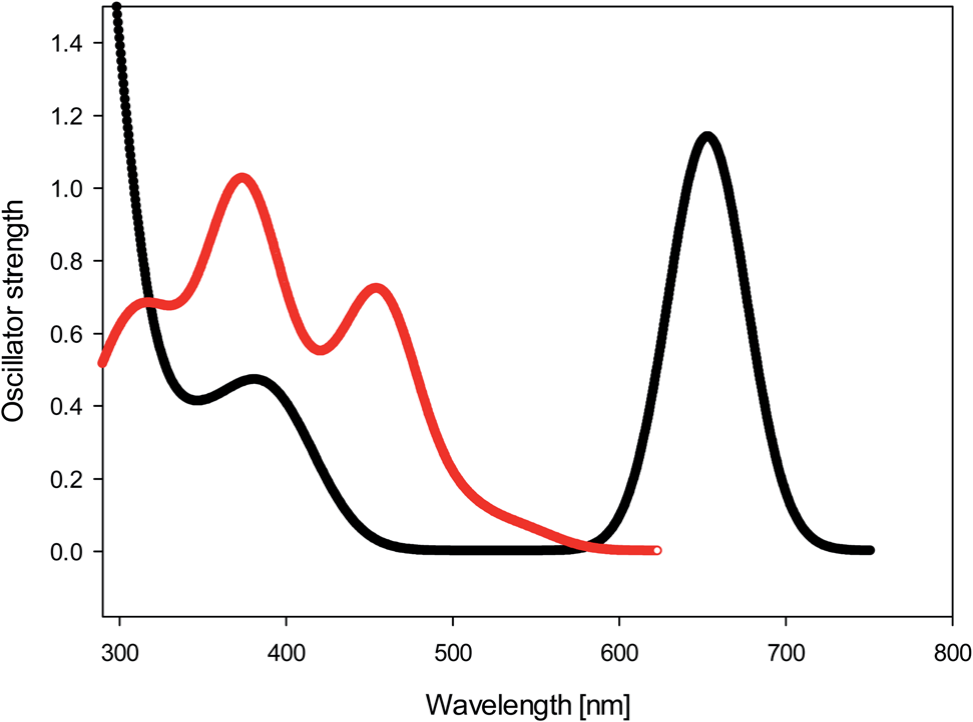
Black: calculated UV-Vis spectrum (TD-B3LYP/6-31+G(d)//M06-2X/cc-pVDZ) of diol 9. Red: calculated UV-Vis spectrum (TD-B3LYP/6-31+G(d)//M06-2X/cc-pVDZ) of bay quinone 10.

We calculated (TD-B3LYP/6-31+G(d)/M06-2X/cc-pVDZ) the UV-Vis spectra of diol 9 and bay quinone 10. The results, shown in Fig. 5, show that the UV-Vis spectrum of precursor 9 is reasonably well reproduced by the DFT calculation. The UV-Vis spectrum of bay quinone 10 is calculated to show absorption in the range 300–500 nm (where 9 is calculated to have only a weak band), which is fully consistent with the experimental spectrum show in Fig. 2, the only difference being that the fine structure of the 300–500 nm band system of 10 is not resolved in the experimental spectrum.

**Fig. 6:**
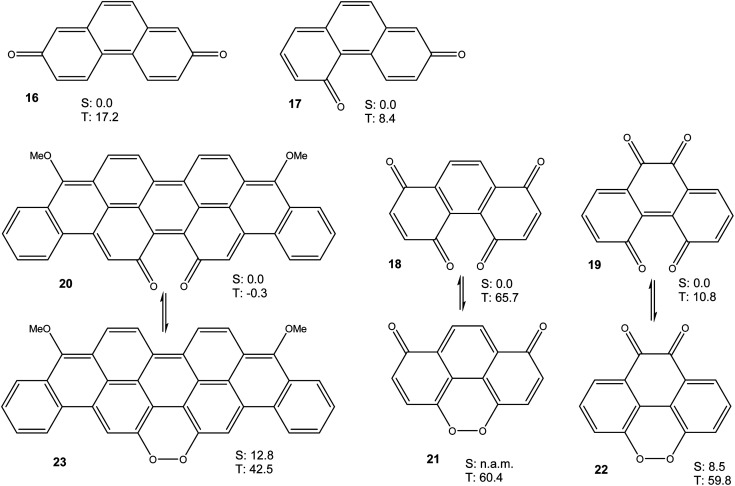
Free energies (M06-2X/cc-pVTZ, in kcal mol^−1^) of lowest singlet and triplet states of 16–23. The reference points are the lowest singlet states of the quinone tautomers 16–20. n.a.m.: not a minimum.

Taken together, the results presented in Fig. 3 and 4 show that a bay quinone moiety, such as in 4 or 10, may result in a biradicaloid system with a very small triplet energy, but it does not necessarily have to. What about bay dicarbonyl moieties in general? Which electronic factors would favour a pronounced biradical character in bay quinones? In order to assess these questions, we performed further DFT calculations on phenanthrene-2,7-quinone 16, phenanthrene-2,5-quinone 17, phenanthrene-1,4,5,8-diquinone 18, phenanthrene-4,5,9,10-diquinone 19, and the reduced violanthrone derivative 20. For 18–20, the isomeric peroxides 21–23 were also investigated. For all compounds, the triplet energy, as a measure of diradical character, was calculated (Fig. 6).

**Fig. 7:**
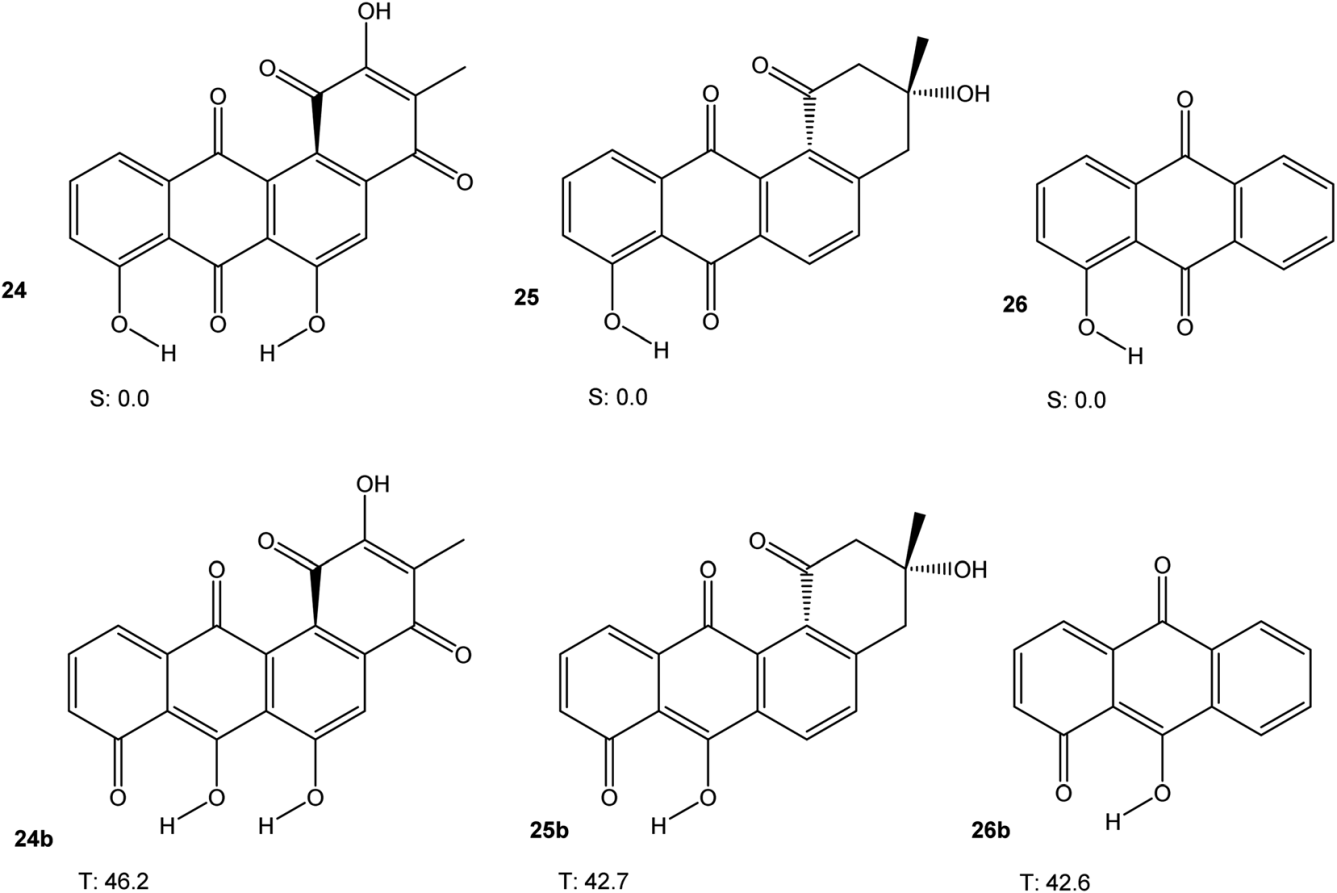
Triplet free energies (M06-2X/cc-pVTZ) of 24, 25, and 26.

Some angucycline antibiotics, like oviedomycin 24 or tetrangomycin 25 show a bay quinone (24) or bay dicarbonyl (25) moiety. In order to assess whether this particular arrangement might influence the properties of 24 and 25, we performed DFT calculations (M06-2X/cc-pVTZ) on 24 and 25, along with hydroxyanthraquinone 26 as a model compound, in their singlet ground and lowest triplet excited states. The results are shown in Fig. 7. Table 1 summarises the results of all calculations.

**Table 1:** Selected parameters (energy, excitation energy, geometric) of a series of quinones or endoperoxides. Optimizations at M06-2X/cc-pVTZ^a^

System	Δ*U* (HOMO–LUMO) [eV]	*λ*_max,calc_ [nm]	Triplet free energy [kcal mol^−1^]	*R*_(O–O)_ [Å]	*Φ*_(C(=O)–C–C–C(=O))_ [°]	*R*_(O–O)_ [Å] (triplet)	*Φ*_(C(=O)–C–C–C(=O))_ (triplet) [°]
4	2.011	695.7^b^	2.1	2.825	19.8	2.644	23.7
13	4.490	345.4^b^	8.2	1.437	10.3	1.985	0.0
16	2.637	572.6^c^	17.2	n.a.	n.a.	n.a.	n.a.
17	2.215	646.8^c^	8.4	n.a.	n.a.	n.a.	n.a.
18	3.932	452.2^c^	65.7	2.726	22.7	2.801	15.8
21	n.a.m.	n.a.m.	n.a.	n.a.m.	n.a.m.	1.990	0.0
19	2.591	613.2^c^	10.8	2.835	19.3	2.648	32.1
22	3.206	547.1^c^	51.3	1.432	11.6	1.424	5.9
9	2.357	653.4^d^	28.9	2.653	30.8	2.671	26.8
10	2.863	542.5^b^	35.5	2.772	23.5	2.600	29.2
15	2.057	685.7^d^	6.5	1.435	10.6	1.990	0.0
20	1.675	1151.6^d^	−0.8	2.703	24.7	2.703	25.0
23	2.579	505.8^d^	29.6	1.441	9.3	1.436	10.5
24	3.371	447.1^c^	46.2	2.699	23.2	2.690	32.4
25	3.622	420.0^c^	42.7	2.786	22.2	2.760	22.2
26	3.597	411.7^c^	42.6	n.a.	n.a.	n.a.	n.a.

a n.a.m.: not a minimum. n.a.: not available.

b TD-B3LYP/6-311++G(2df,p)//M06-2X/cc-pVTZ.

c TD-B3LYP/6-311++G(d,p)//M06-2X/cc-pVTZ.

d TD-B3LYP/6-31+G(d)//M06-2X/cc-pVTZ.

Taken together, the results presented in Fig. 3, 4, 6 and 7 indicate that aromatic stabilisation likely is the crucial factor in determining the stability and biradical character of the quinones and cyclic peroxides investigated. Triplet energies and therefore biradical character correlate with the number of Clar sextets available.^15^*E.g.*, in the case of 4/10, with zero Clar sextets for 4, and two for 10, the free energy of the peroxide tautomer (which, with its unfavourable O–O bond would normally be very high in energy), is only slightly above the free energy of the quinone. A similar observation is made for 19/22. In both cases, the quinone (4 or 19) also has a very low triplet free energy, indicating a large degree of biradical character. If, on the other hand, the quinone has an unambiguous Clar sextet, as in 18, the triplet energy calculated is significant. In this case, the peroxide tautomer 21 (which does not have any Clar sextets) is calculated to be a minimum structure only on the triplet potential energy hypersurface. A geometry optimisation on the singlet PES resulted in optimisation of quinone 18.^16^ Considering quinone 20/peroxide 23, it is the peroxide tautomer 23 that contains the larger number of Clar sextets (20 : 2; 23 : 4). Consistent with this, the energy difference between the quinone tautomer and the peroxide tautomer 23 here is calculated to be smaller than for all other systems calculated. Remarkably, quinone 20 is predicted to have lowest triplet and open-shell lowest singlet states that are energetically essentially degenerate (in terms of *U*, the singlet state is slightly lower in energy, while the inclusion of entropy leads to a triplet ground state in terms of *G*).

Quinones 4, 16 and 17 all have the same number of Clar sextets (zero). Correspondingly, they all have fairly low triplet energies, yet there is some variation. A comparison of the triplet energies of 4, 16 and 17 reveals that they correlate with the number of carbonyl groups in bay positions, the triplet energy being the lowest when both carbonyl groups are in bay positions, as in 4.

The discussion of the triplet free energies of the angucyclines investigated is complicated by the fact that optimisation of the triplet states in each case resulted in convergence of the geometry optimisation to a product in which one phenolic hydrogen had shifted its position (as indicated by the letter “b”). Nevertheless, the results suggest that a bay quinone or -dicarbonyl moiety does not modify the electronic properties of these angucyclines in a way that would confer significant biradical-type reactivity.

## Conclusion

The presence of a bay dicarbonyl moiety or a bay quinone in a molecule may, but does not have to, confer significant biradical character. As far as quinones are concerned, aromaticity (*e.g.*, as expressed by the number of Clar sextets present) appears to be the single most dominant criterion determining the triplet energy and thus biradical character. For isomeric quinones with equal number of Clar sextets, the presence of one or both carbonyl moieties in bay positions then additionally reduces the triplet energy. Among the molecules investigated in this study, this is most prominent for phenanthrene-4,5-quinone, which is predicted to have a very small triplet free energy of only Δ*G*_triplet_ = 2.1 kcal mol^−1^. Bay quinones may have peroxide valence tautomers. These cyclic peroxides normally are energetically well above the bay quinone tautomers. However, if the peroxides are more aromatic than the quinones, this energy difference is reduced. Under certain conditions (relatively large π-system, peroxide tautomer more aromatic than bay quinone tautomer), the bay quinone may also have a triplet ground state. Finally, the presence of a bay quinone or bay dicarbonyl moiety in some angucycline natural products is not expected to have significant impact on the electronic properties of these quinoid compounds.

## Experimental part

**Scheme 3:**
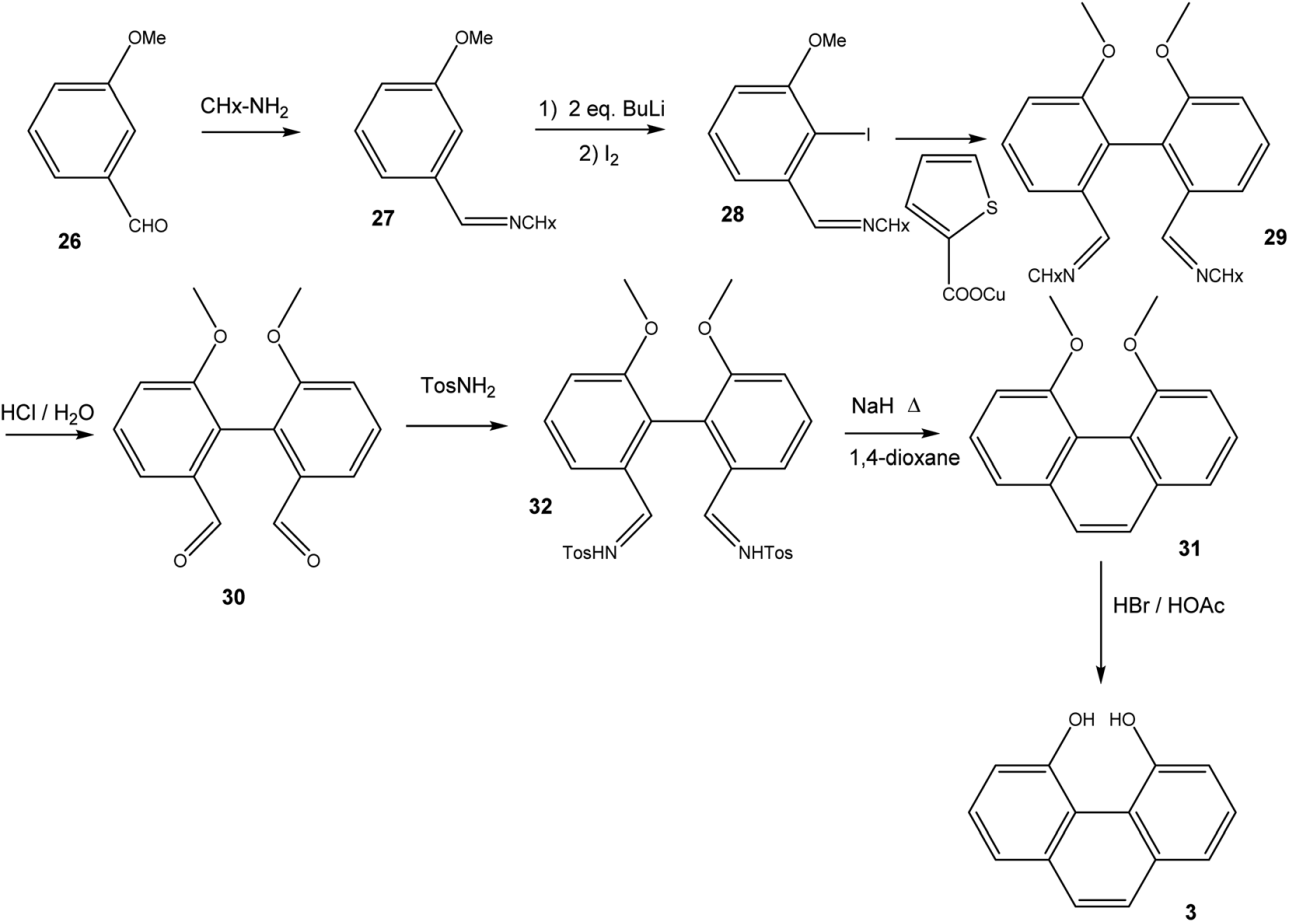
Synthesis of 4,5-dihydroxyphenanthrene 3.

16,17-Dihydroxyviolanthrone 9 was obtained from Sigma-Aldrich and was used (with identical results) both as received and after column filtration over SiO_2_ using DMF as eluent. The synthesis of 4,5-dihydroxyphenanthrene 3 (Scheme 3) initially followed the route to 2,2′-dimethoxy-biphenyl-6,6′-dialdehyde-dicyclohexylimine 29, as published by Breit and Reichert.^17^ Thus, 3-methoxybenzaldehyde 26 was converted to imine 27, which was iodinated in 2-position by treatment with 2 eq. *n*-BuLi followed by quenching of the organolithium compound with iodine. Iodoarene 28 was then homocoupled under Ullmann-conditions, employing copper(i) thiophene-2-carboxylate^18^ as coupling reagent. Acid hydrolysis of biphenyldialdimine 29 then yielded dialdehyde 30. In order to convert 30 into 4,5-dimethoxyphenanthrene 31, we followed the synthetic protocol by Jung and Hagiwara, converting 30 into the bistosylhydrazone 32, after which a Bamford–Stevens reaction in refluxing 1,4-dioxane yielded 31 in fairly low yield.^19^ Finally, 31 was demethylated using HBr/HOAc.

### 6,6′-Dimethoxybiphenyl-2,2′-dicarbaldehyde 30

To a solution of 28 (ref. 17) (7.5 g, 21.8 mmol) in NMP (50 mL) was added copper(i)-thiophenecarboxylate (10.4 g, 54.5 mmol, 2.5 eq.). The resulting suspension was stirred for 24 h at room temperature with exclusion of light. Addition of conc. aqueous NH_3_ (250 mL) resulted in a blue solution, which was extracted with EtOAc (4 × 100 mL). The combined organic layers were washed with aqueous ammonia solution (3 × 50 mL, 12.5%), brine (2 × 50 mL) The organic phase was dried over MgSO_4_ and the solvent was removed under vacuum yielding crude residue of 29. The residue 29 was dissolved in DCM (50 mL) and HCl (35 mL, 6 M) was added. The mixture was stirred at room temperature for 2 h. The layers were separated and the aqueous phase was extracted with DCM (3 × 40 mL). The combined organic layers were washed with HCl (2 × 20 mL, 1 M), sat. aqueous NaHCO_3_ (40 mL) and brine (40 mL) The organic phase was dried over MgSO_4_ and the solvent was removed under vacuum. The residue was purified by column chromatography (hexane/EtOAc 5 : 1 → 1 : 1) yielding a white solid 30 (0.8 g, 3.1 mmol, 28% yield). ^1^H NMR (400 MHz, CDCl_3_): *δ* 3.76 (s, 6H), 7.25 (dd, *J* = 0.9,8.2, 2H), 7.57 (t, *J* = 8.0, 2H), 7.70 (dd, *J* = 1.0,7.9, 2H), 9.69 (s, 2H), [impurity *δ* 7.28, chloroform]; ^13^C NMR (100 MHz, CDCl_3_): *δ* 56.0 (2C), 115.9 (2C), 119.8 (2C), 125.5 (2C), 129.8 (2C), 135.9 (2C), 157.2 (2C), 191.7 (2C); mp: 110 °C.

### 6,6′-Dimethoxybiphenyl-2,2′-dicarbaldehyde-bis-tosylhydrazone 32 (ref. 19)

Dialdehyde 30 (0.51 g, 1.9 mmol) was dissolved in methanol (1 mL) and added dropwise to a rapidly stirred suspension of *p*-toluenesulfonyl hydrazide (0.88 g, 4.75 mmol, 2.5 eq. excess) in methanol (3 mL). A mildly exothermic reaction ensued and the hydrazide dissolved. After 10 min the bis-tosylhydrazone began to precipitate. After 25 min the reaction was cooled to 0 °C and the product removed by filtration and washed with methanol (3 mL). The product was dried under vacuum to remove any residual methanol. The crude product 32 was recrystallized from methanol yielding 0.67 g (1.11 mmol), 58% yield. ^1^H NMR (400 MHz, (CD_3_)_2_SO): *δ* 2.37 (s, 6H), 2.51 (m, 2H), 3.52 (s, 6H), 7.09 (d, *J* = 5.8 Hz, 4H), 7.10 (d, *J* = 5.8 Hz, 4H), 7.29 (s, 2H), 7.37–7.41 (m, 4H), 7.67 (d, *J* = 8.3 Hz, 2H), ^13^C NMR (100 MHz, (CD_3_)_2_SO)): *δ* 21.5, 56.0, 112.8, 117.1, 123.9, 127.6 (2C), 129.7, 130.2 (2C), 134.0, 136.6, 143.9, 145.5, 157.3; mp: 225 °C (sharp).

### 4,5-Dimethoxyphenanthrene 31

Sodium hydride (0.07 g, 2.70 mmol, 2.4 eq) was added to 32 (0.67 g 1.11 mmol) in anhydrous 1,4-dioxane (10 mL). The mixture was stirred at 2 h under argon then heated to 120 °C and set to reflux for 12 h. The reaction mixture was cooled to room temperature then ethanol (10 mL) and water (200 mL) were added. The solution was extracted with DCM (5 × 50 mL). The organic phase was washed with water (2 × 50 mL) then dried over MgSO_4_. The solvent was removed under vacuum yielding a white solid (0.05 g, 0.20 mmol, 18% yield). ^1^H NMR (400 MHz, CDCl_3_): *δ* 3.96 (s, 6H), 7.03 (dd, *J* = 1.0, 8.0 Hz, 2H), 7.34 (dd, *J* = 1.1, 8.0 Hz, 2H), 7.43 (t, *J* = 7.8, 2H), 7.49 (s, 2H) ^13^C NMR (400 MHz, CDCl_3_): *δ* 55.8 (2C), 108.3 (2C), 119.2 (2C), 119.7 (2C), 126.6 (2C), 126.8 (2C), 134.6 (2C), 157.8 (2C).

### 4,5-Dihydroxyphenanthrene 3

31 (0.22 g, 0.93 mmol) was added to a HBr solution (5 mL of 33 wt% in acetic acid) and set to reflux at 120 °C. The reaction was monitored using TLC and after 2 h the reaction was complete. The reaction was cooled to room temperature then quenched with saturated Na_2_CO_3_ (50 mL), neutralising the acid. The solution was extracted with DCM (3 × 50 mL). The organic phase was dried over MgSO_4_ then the solvent was removed under vacuum. The crude material was then purified using column chromatography (SiO_2_, DCM) yielding a grey powder (0.09 g, 0.44 mmol, 47% yield). ^1^H NMR (400 MHz, CDCl_3_): *δ* 7.0–7.51 (m, 8H), 10.08 (broad s, 2H) ^13^C NMR (100 MHz, CDCl_3_): *δ* 115.2 (2C), 118.5 (2C), 121.9 (2C), 127.3 (2C), 127.7 (2C), 135.2 (2C), 151.4 (2C); mp: 160–163 °C.

### Electrochemistry and UV-Vis spectroscopy

All solvents for electrochemistry were obtained from Sigma Aldrich and used as supplied. Tetrabutylammonium hexafluorophosphate (TBA-PF_6_) (99%) was supplied by Sigma Aldrich. Electrochemical studies were performed in a single chamber cell in a three-electrode configuration using a CH Instruments CHI700 series potentiostat. The supporting electrolyte was 0.1 M TBA-PF_6_ in dimethylformamide, unless otherwise noted. A Pt wire was used as the counter electrode, along with an Ag/AgNO_3_ pseudo reference electrode. Potentials are reported relative to the ferrocenium/ferrocene couple, the position of which was judged by adding ferrocene to the samples analyzed. Working electrodes were washed with acetone and deionized water prior to use. Cyclic voltammograms were collected at room temperature under an atmosphere of Ar at a scan rate of 100 mV s^−1^ (unless otherwise stated). A glassy carbon button electrode (area = 0.071 cm^2^, CH Instruments) was used as the working electrode for cyclic voltammetry. Measurements were conducted without stirring and with *iR* compensation enabled. Bulk electrolyses were carried out in 0.1 M TBA-PF_6_ in dimethylformamide using an Ag/AgNO_3_ pseudo reference electrode, a Pt wire counter electrode and a large area carbon felt working electrode. Solutions were stirred during bulk electrolysis. Room temperature was 20 °C in these studies. Temperatures of −44 °C were achieved by immersing the cell in a dry-ice/acetonitrile bath. UV-Vis spectra were recorded on a JASCO V-670 spectrophotometer using 1 cm path length cuvettes.

### Computational methods

All calculations were performed employing the Gaussian 09 suite of programmes.^20^ All stationary points were characterised as minima or transition states by performing a vibrational analysis. DFT calculations were performed employing the M06-2X hybrid functional,^21^ employing cc-pVDZ or cc-pVTZ basis sets,^22^ or using the B3LYP method.^23^ UV-Vis spectra were calculated at the TD-B3LYP^24^ level of theory with a 6-31+G(d), 6-311++G(d,p) or 6-311++G(2df,p)^25^ basis set.

## Conflicts of interest

There are no conflicts to declare.

## Supplementary Material

RA-010-D0RA06519F-s001
