# Supplementary material for: Attempted characterisation of phenanthrene-4,5-quinone and electrochemical synthesis of violanthrone-16,17-quinone. How does the stability of bay quinones correlate with structural and electronic parameters?
Source: RSC Adv. 2020 Oct 15;10(62):38004–12. doi: 10.1039/d0ra06519f (PMC9057194; doi:10.1039/d0ra06519f)
Supplement: RA-010-D0RA06519F-s001 [file RA-010-D0RA06519F-s001.pdf]

Electronic Supplementary Material (ESI) for RSC Advances.
This journal is © The Royal Society of Chemistry 2020

Bay Quinones: Attempted Characterisation of Phenanthrene-4,5-Quinone and Electrochemical Synthesis of Violanthrone-16,17-Quinone

Dylan Wilkinson, Giacomo Cioncoloni, Mark D. Symes, and Götz Bucher*

School of Chemistry, University of Glasgow, University Avenue, Glasgow G12 8QQ, United Kingdom.
E-Mail: goetz.bucher@glasgow.ac.uk

Supporting Information

Figures S1-S3 (electrochemistry): S2

Calculated UV/Vis spectrum of 4 S5

Cartesian coordinates and electronic energies S6

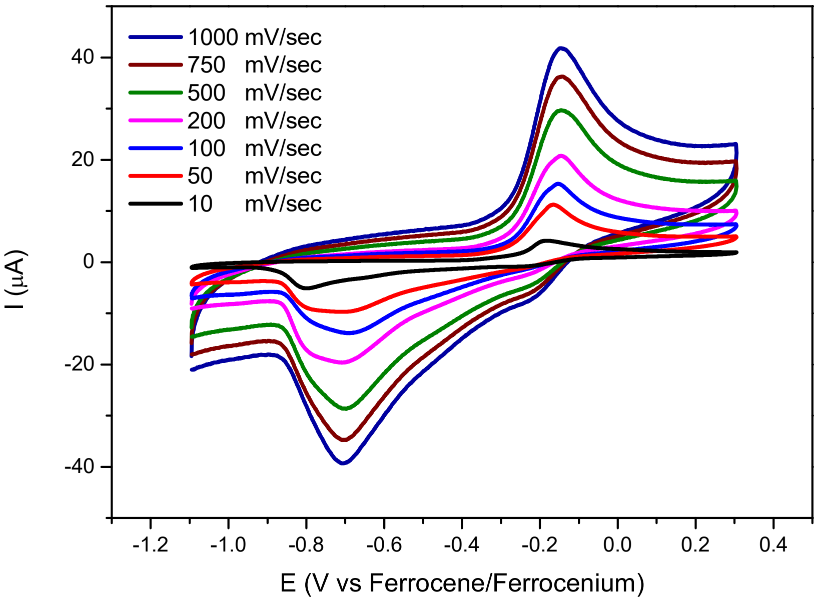

Figure S1: Scan-rate-dependency study for a 3 mM solution of 16,17-dihydroxyviolanthrone in dimethyl formamide / 0.1 M $TBAPF_6$ at room temperature.

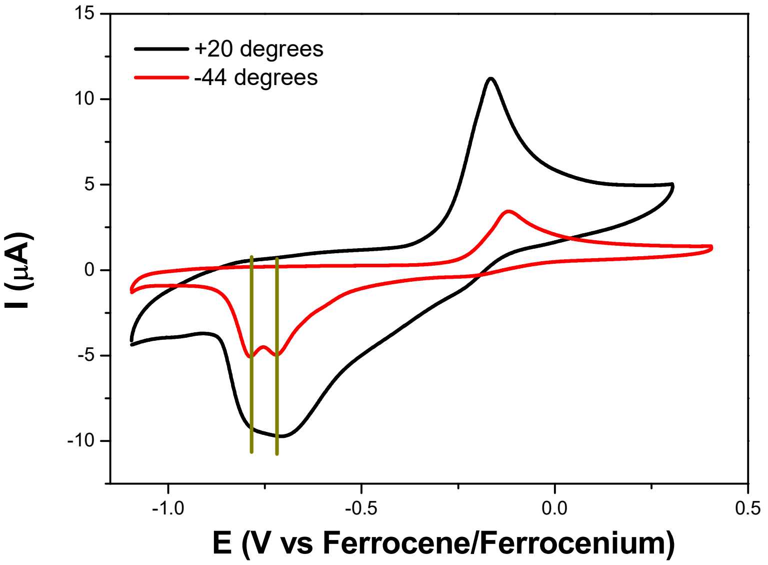

Figure S2: Comparison between the cyclic voltammograms of a 3 mM solution of 16,17-dihydroxyviolanthrone in dimethyl formamide / 0.1 M $TBAPF_6$ at room temperature (black trace) and at −40 °C (red trace). The scan-rate in both cases was 50 mVs^{-1}.

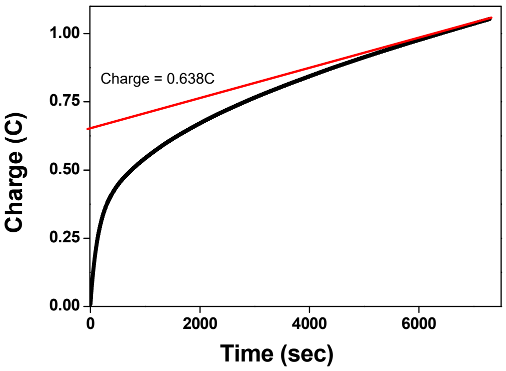

Figure S3a: A representative charge *vs.* time curve for the bulk electrolysis (run according to the conditions in the Experimental Section) of a 0.24 mM solution of 16,17-dihydroxyviolanthrone in 15 mL dimethylformamide / 0.1 M $TBAPF_6$ at room temperature at a potential of 0 V *vs.* ferrocenium/ferrocene. The red dashed line shows the regression from the period of constant current at t > 5000 s to determine the charge passed in the performing the oxidation event. This corresponds to 638 mC. On the basis of the amount of compound in solution, 347 mC is expected for a 1-electron process. The amount of charge observed therefore corresponds to 92% of the charge expected for a 2-electron process.

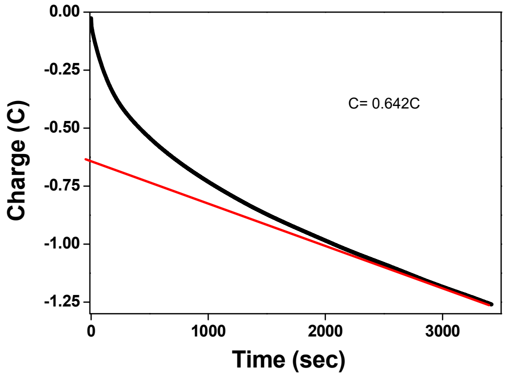

Figure S3b: A charge *vs.* time curve for the reductive bulk electrolysis of the oxidised sample generated during the experiment shown in Figure S3a. The reduction was performed at −1 V *vs.* ferrocenium/ferrocene. The red dashed line again shows the regression from the period of constant current at t > 2500 s to determine the charge passed in the performing this reduction process. This corresponds to 642 mC, in good agreement with the charge required to oxidise this sample.

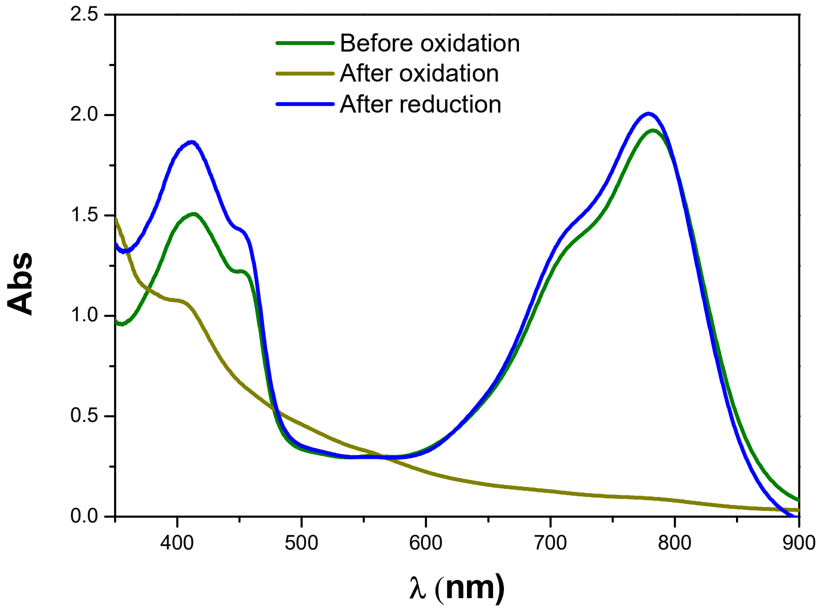

Figure S4: Comparison between the spectra of fresh 16,17-dihydroxyviolanthrone (approximately 0.3 mM, green), the same sample after electrochemical 2-electron oxidation (khaki), and the same sample after electrochemical re-reduction by two electrons (blue). Note that the concentration of the fresh and re-reduced samples is slightly different.

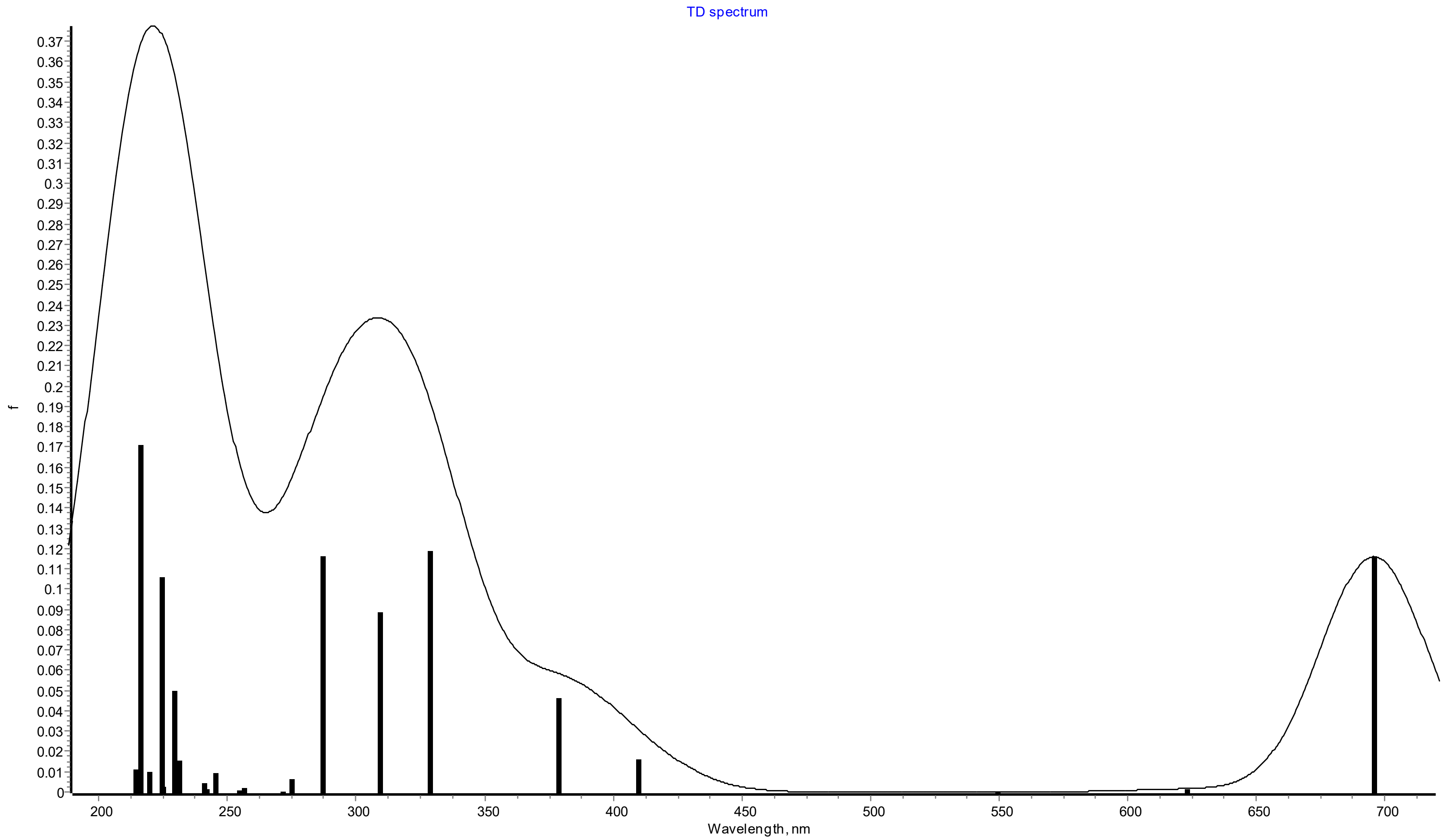

Figure S5: Calculated spectrum (TD-B3LYP/6-311++G(2df,p)//M06-2X/cc-pVTZ) of quinone **4**.

Cartesian coordinates and electronic energies of stationary points optimized

n.b.: all stationary points have exactly zero imaginary frequencies (if minima), or exactly one imaginary frequency (if transition states)

Phenanthrene-4,5-quinone 4, M06-2X/cc-pVTZ

1	-4.557577000	-0.004765000	-0.420525000
6	-3.492834000	-0.046436000	-0.231163000
1	-3.306384000	2.127135000	-0.286363000
6	-2.762933000	1.198193000	-0.171137000
6	-1.469531000	-1.265157000	0.353892000
6	-1.414208000	1.233648000	-0.020713000
6	-2.890165000	-1.227961000	-0.026579000
6	-0.675478000	-0.018250000	0.062455000
6	-0.669388000	2.473706000	-0.022960000
1	-3.425771000	-2.166056000	-0.005019000
6	0.669388000	2.473706000	0.022960000
1	-1.228275000	3.399987000	-0.053081000
6	1.414208000	1.233648000	0.020713000
6	2.762932000	1.198193000	0.171137000
6	0.675478000	-0.018250000	-0.062455000
6	3.492833000	-0.046436000	0.231164000
6	2.890165000	-1.227961000	0.026579000
1	3.425771000	-2.166056000	0.005020000
6	1.469531000	-1.265156000	-0.353892000
8	-1.005284000	-2.180346000	0.992599000
8	1.005284000	-2.180345000	-0.992599000
1	4.557576000	-0.004765000	0.420526000
1	1.228274000	3.399987000	0.053081000
1	3.306383000	2.127135000	0.286364000

E(M06-2X): -688.652221

Phenanthrene-4,5-quinone 4, lowest triplet excited state, UM06-2X/cc-pVTZ

1	0.271928000	4.639391000	0.088975000
6	0.158068000	3.564908000	0.046699000
1	0.292001000	3.310110000	2.171368000
6	0.181674000	2.822014000	1.212154000
6	-0.297859000	1.507756000	-1.248124000
6	0.076747000	1.396399000	1.189422000
6	-0.064688000	2.935856000	-1.160246000
6	-0.019996000	0.707075000	-0.031323000
6	0.064688000	0.677596000	2.408734000
1	-0.173736000	3.487957000	-2.083154000
6	-0.064688000	-0.677596000	2.408734000
1	0.128463000	1.226582000	3.339281000
6	-0.076747000	-1.396399000	1.189422000

6	-0.181674000	-2.822014000	1.212154000
6	0.019996000	-0.707075000	-0.031323000
6	-0.158068000	-3.564908000	0.046699000
6	0.064688000	-2.935856000	-1.160246000
1	0.173736000	-3.487957000	-2.083154000
6	0.297859000	-1.507756000	-1.248124000
8	-0.821679000	1.035713000	-2.252546000
8	0.821679000	-1.035713000	-2.252546000
1	-0.271928000	-4.639391000	0.088975000
1	-0.128463000	-1.226582000	3.339281000
1	-0.292001000	-3.310110000	2.171368000

E(M06-2X): -688.6462523

Transition state for conversion of phenanthrene-4,5-quinone 4 into oxirane 11, M06-2X/cc-pVTZ

1	4.106880000	0.240680000	-1.143413000
6	3.108563000	0.177034000	-0.734472000
1	2.942791000	2.322588000	-0.603544000
6	2.450133000	1.383354000	-0.375589000
6	1.311592000	-1.130033000	0.322416000
6	1.159315000	1.408908000	0.142361000
6	2.525097000	-1.047602000	-0.560280000
6	0.555648000	0.157626000	0.292765000
6	0.269599000	2.548799000	0.189303000
1	3.048564000	-1.973956000	-0.745453000
6	-1.081812000	2.394061000	0.220462000
1	0.696825000	3.542613000	0.145637000
1	-1.721987000	3.266249000	0.230211000
6	-1.688591000	1.086418000	0.135628000
6	-3.016882000	0.742695000	-0.057295000
6	-0.782626000	0.003881000	0.175096000
6	-3.340376000	-0.611433000	-0.269569000
1	-3.795384000	1.491780000	-0.083156000
1	-4.384285000	-0.850709000	-0.432158000
6	-2.430999000	-1.662819000	-0.331447000
1	-2.753951000	-2.668222000	-0.555136000
6	-1.076255000	-1.364526000	-0.126763000
8	1.795365000	-1.621174000	1.369358000
8	-0.034601000	-2.114977000	-0.237944000

E(M06-2X): -688.6042564

Transition state for conversion of phenanthrene-4,5-quinone 4 into oxirane 11, triplet PES, UM06-2X/cc-pVTZ

1	4.210325000	0.111251000	-0.946627000
6	3.190765000	0.104832000	-0.583376000
1	3.160990000	2.262052000	-0.424960000
6	2.597313000	1.351195000	-0.278984000
6	1.279331000	-1.150140000	0.259077000
6	1.232890000	1.395519000	0.095650000

6	2.571341000	-1.112436000	-0.427957000
6	0.614004000	0.171101000	0.248764000
6	0.365732000	2.543479000	0.150613000
1	3.094564000	-2.041519000	-0.600663000
6	-0.995259000	2.422470000	0.209726000
1	0.809723000	3.529825000	0.105836000
1	-1.609997000	3.312825000	0.234031000
6	-1.628265000	1.136488000	0.143003000
6	-2.987053000	0.809400000	-0.036595000
6	-0.754177000	0.052596000	0.153903000
6	-3.351173000	-0.509130000	-0.257828000
1	-3.743575000	1.582495000	-0.031005000
1	-4.398619000	-0.733329000	-0.409259000
6	-2.433820000	-1.578602000	-0.345859000
1	-2.760859000	-2.575668000	-0.599894000
6	-1.101183000	-1.275860000	-0.167745000
8	1.237533000	-1.898586000	1.346118000
8	-0.033186000	-2.053088000	-0.383843000

E(M06-2X): -688.5761737

Oxirane 11, M06-2X/cc-pVTZ

1	4.138801000	0.243165000	-0.973942000
6	3.142526000	0.216115000	-0.553149000
1	3.054870000	2.311217000	-0.386847000
6	2.527162000	1.376555000	-0.243366000
6	1.118264000	-1.118815000	0.134542000
6	1.117516000	1.411493000	0.117769000
6	2.519705000	-1.117255000	-0.383475000
6	0.511823000	0.196804000	0.241371000
6	0.218238000	2.523998000	0.110628000
1	2.892602000	-1.953864000	-0.957641000
6	-1.152557000	2.366746000	0.130906000
1	0.624988000	3.525529000	0.046880000
1	-1.780426000	3.247867000	0.103035000
6	-1.759273000	1.072488000	0.109475000
6	-3.105606000	0.657304000	-0.057400000
6	-0.849033000	0.027753000	0.171374000
6	-3.382509000	-0.685165000	-0.207249000
1	-3.909034000	1.380027000	-0.097160000
1	-4.414196000	-0.980324000	-0.345590000
6	-2.403583000	-1.722763000	-0.230500000
1	-2.688025000	-2.748125000	-0.411950000
6	-1.112426000	-1.332597000	-0.032033000
8	2.149721000	-1.485007000	0.966484000
8	0.067645000	-2.047675000	-0.070251000

E(M06-2X): -688.6694721

Transition state for conversion of oxirane 11 into ketone 12, M06-2X/cc-pVTZ

6	-1.918434000	0.960566000	0.048127000
6	-2.324994000	-1.899721000	-0.030631000
6	-0.929637000	0.011641000	-0.102257000
6	-3.222930000	0.415167000	0.140781000
6	-3.388274000	-0.957900000	0.095406000
6	-1.073893000	-1.364954000	-0.130020000
1	-4.089262000	1.052276000	0.256406000
1	-4.390615000	-1.355058000	0.173236000
1	-2.522979000	-2.961240000	-0.032451000
8	0.205123000	-1.933867000	-0.229396000
6	-1.398055000	2.295069000	0.116824000
1	-2.085948000	3.123307000	0.235528000
6	-0.041765000	2.576724000	0.062176000
1	0.274197000	3.607867000	0.146509000
6	0.948902000	1.556622000	-0.077614000
6	0.417129000	0.287735000	-0.182734000
6	1.085896000	-0.932863000	-0.251488000
6	2.392841000	1.620801000	-0.064963000
1	2.888735000	2.575281000	0.052895000
6	3.091278000	0.463121000	-0.121418000
1	4.171759000	0.485228000	-0.047706000
6	2.576662000	-0.993666000	-0.193319000
1	2.884713000	-1.291307000	-1.248368000
8	2.992508000	-1.749432000	0.805737000

E(M06-2X): -688.588985

Ketone 12, M06-2X/cc-pVTZ

6	1.880277000	0.967597000	-0.029472000
6	2.271590000	-1.896083000	-0.063757000
6	0.899805000	0.020446000	0.245358000
6	3.168079000	0.416474000	-0.257578000
6	3.323977000	-0.952923000	-0.257382000
6	1.035682000	-1.373164000	0.181046000
1	4.016485000	1.055842000	-0.458704000
1	4.311045000	-1.352171000	-0.450381000
1	2.455791000	-2.956485000	-0.144114000
8	-0.187458000	-1.980666000	0.314519000
6	1.392045000	2.304861000	-0.116017000
1	2.084668000	3.113254000	-0.309746000
6	0.041749000	2.583825000	-0.033431000
1	-0.290624000	3.603049000	-0.185684000
6	-0.929026000	1.566795000	0.199919000
6	-0.427473000	0.320996000	0.417965000
6	-1.156804000	-0.954901000	0.596495000
6	-2.379795000	1.606826000	0.018573000
1	-2.880337000	2.563710000	-0.074531000
6	-3.083804000	0.469019000	-0.139909000
1	-4.136861000	0.495084000	-0.384341000
6	-2.468101000	-0.899334000	-0.177979000

1	-1.480909000	-1.064793000	1.642855000
8	-2.998600000	-1.836847000	-0.706812000

E(M06-2X): -688.6924186

Transition state for conversion of quinone 4 into peroxide 13, M06-2X/cc-pVTZ

1	4.429944000	0.009949000	0.794864000
6	3.388284000	0.008521000	0.503770000
1	3.317477000	2.141405000	0.235833000
6	2.765516000	1.212558000	0.177776000
6	1.402520000	-1.216237000	-0.091099000
6	1.414577000	1.242763000	-0.145187000
6	2.699410000	-1.186023000	0.468254000
6	0.705347000	0.023069000	-0.282430000
6	0.673912000	2.470874000	-0.270840000
1	3.170408000	-2.129069000	0.703083000
6	-0.673912000	2.470874000	-0.270840000
1	1.225705000	3.401796000	-0.289535000
6	-1.414577000	1.242763000	-0.145187000
6	-2.765516000	1.212558000	0.177776000
6	-0.705347000	0.023069000	-0.282430000
6	-3.388284000	0.008521000	0.503770000
6	-2.699410000	-1.186023000	0.468254000
1	-3.170408000	-2.129069000	0.703083000
6	-1.402520000	-1.216237000	-0.091099000
8	0.914322000	-2.344653000	-0.450713000
8	-0.914322000	-2.344653000	-0.450713000
1	-4.429944000	0.009949000	0.794864000
1	-1.225705000	3.401796000	-0.289535000
1	-3.317477000	2.141405000	0.235834000

E(M06-2X): -688.6112032

Peroxide 13, optimized geometry , M06-2X/cc-pVTZ

1	0.000163000	4.545284000	-0.085446000
6	-0.002300000	3.465098000	-0.038153000
1	0.108394000	3.417689000	2.095233000
6	0.065777000	2.837545000	1.183037000
6	-0.089466000	1.369729000	-1.170214000
6	0.060526000	1.430248000	1.254893000
6	-0.089466000	2.736962000	-1.239850000
6	0.010825000	0.709739000	0.058198000
6	0.041471000	0.676774000	2.477776000
1	-0.177405000	3.232397000	-2.195763000
6	-0.041471000	-0.676774000	2.477776000
1	0.073997000	1.218576000	3.414405000
1	-0.073997000	-1.218576000	3.414405000
6	-0.060526000	-1.430248000	1.254893000
6	-0.065777000	-2.837545000	1.183037000

6	-0.010825000	-0.709739000	0.058198000
6	0.002300000	-3.465098000	-0.038153000
1	-0.108394000	-3.417689000	2.095233000
1	-0.000163000	-4.545284000	-0.085446000
6	0.089466000	-2.736962000	-1.239850000
1	0.177405000	-3.232397000	-2.195763000
6	0.089466000	-1.369729000	-1.170214000
8	-0.355952000	0.623955000	-2.297819000
8	0.355952000	-0.623955000	-2.297819000

E(M06-2X): -688.652221

Peroxide 13, lowest triplet excited state, UM06-2X/cc-pVTZ

1	-0.113024000	4.622813000	0.000000000
6	-0.068323000	3.542054000	0.000000000
1	-2.210251000	3.302830000	0.000000000
6	-1.253139000	2.799143000	0.000000000
6	1.233908000	1.495787000	0.000000000
6	-1.223878000	1.415928000	0.000000000
6	1.146478000	2.915410000	0.000000000
6	0.018879000	0.706490000	0.000000000
6	-2.453054000	0.670824000	0.000000000
1	2.077079000	3.463233000	0.000000000
6	-2.453054000	-0.670824000	0.000000000
1	-3.381147000	1.227385000	0.000000000
1	-3.381147000	-1.227385000	0.000000000
6	-1.223878000	-1.415929000	0.000000000
6	-1.253139000	-2.799143000	0.000000000
6	0.018879000	-0.706491000	0.000000000
6	-0.068323000	-3.542053000	0.000000000
1	-2.210252000	-3.302830000	0.000000000
1	-0.113024000	-4.622813000	0.000000000
6	1.146478000	-2.915410000	0.000000000
1	2.077078000	-3.463233000	0.000000000
6	1.233908000	-1.495787000	0.000000000
8	2.402764000	0.992453000	0.000000000
8	2.402765000	-0.992454000	0.000000000

E(M06-2X): -688.627255

16,17-Dihydroxyviolanthrone 9, M06-2X/cc-pVTZ

6	-5.738255000	0.401969000	-0.095010000
6	-7.190161000	-1.922517000	0.283943000
6	-5.053559000	-0.794457000	0.153181000
6	-7.130463000	0.429558000	-0.145867000
6	-7.859987000	-0.725458000	0.044721000
6	-5.810403000	-1.956717000	0.335604000
1	-7.610102000	1.378863000	-0.339303000
1	-8.940020000	-0.703880000	0.004917000
1	-5.322592000	-2.903803000	0.511109000

1	-7.750519000	-2.836421000	0.427328000
6	-3.581700000	-0.805857000	0.203905000
6	-0.724474000	-0.851613000	0.108395000
6	-2.855439000	0.397492000	-0.046648000
6	-2.869497000	-1.924145000	0.540248000
6	-1.463234000	-1.949043000	0.524104000
6	-1.438360000	0.379214000	-0.034084000
1	-3.351310000	-2.843075000	0.838747000
6	0.724472000	-0.851616000	-0.108369000
6	3.581699000	-0.805861000	-0.203897000
6	1.438361000	0.379212000	0.034096000
6	1.463230000	-1.949052000	-0.524067000
6	2.869493000	-1.924153000	-0.540222000
6	2.855438000	0.397489000	0.046656000
1	3.351304000	-2.843085000	-0.838716000
6	5.053558000	-0.794459000	-0.153194000
6	7.859988000	-0.725453000	-0.044777000
6	5.810404000	-1.956714000	-0.335646000
6	5.738256000	0.401966000	0.095001000
6	7.130464000	0.429558000	0.145839000
6	7.190162000	-1.922509000	-0.284008000
1	5.322593000	-2.903798000	-0.511160000
1	7.610104000	1.378862000	0.339281000
1	7.750521000	-2.836410000	-0.427419000
1	8.940022000	-0.703872000	-0.004991000
6	-3.527999000	1.617416000	-0.269584000
6	-2.812737000	2.782167000	-0.438432000
1	-3.357172000	3.697974000	-0.621726000
6	-1.426902000	2.778152000	-0.338363000
1	-0.896918000	3.712335000	-0.446334000
6	-0.726394000	1.605057000	-0.106938000
6	0.726395000	1.605057000	0.106942000
6	1.426905000	2.778152000	0.338359000
1	0.896922000	3.712337000	0.446322000
6	2.812740000	2.782166000	0.438430000
1	3.357176000	3.697973000	0.621722000
6	3.528000000	1.617413000	0.269588000
6	5.008375000	1.675466000	0.316369000
8	5.595841000	2.715706000	0.529827000
6	-5.008374000	1.675472000	-0.316359000
8	-5.595839000	2.715715000	-0.529801000
8	-0.930151000	-3.125174000	0.945895000
8	0.930146000	-3.125189000	-0.945838000
1	0.019416000	-3.046271000	1.085323000
1	-0.019422000	-3.046292000	-1.085256000

E(M06-2X): -1606.1904312

Violanthrone-16,17-quinone 10, M06-2X/cc-pVTZ

6	5.725544000	0.337134000	0.003872000
6	7.140316000	-2.028095000	0.212584000
6	5.026986000	-0.874019000	-0.003742000

6	7.115222000	0.361472000	0.100709000
6	7.825831000	-0.816332000	0.202019000
6	5.763097000	-2.056911000	0.111376000
1	7.607647000	1.323723000	0.098348000
1	8.903866000	-0.799798000	0.279548000
1	5.261293000	-3.012300000	0.133810000
1	7.685945000	-2.957330000	0.303434000
6	3.554062000	-0.885589000	-0.128763000
6	0.673379000	-0.803120000	-0.116097000
6	2.833762000	0.396379000	-0.113119000
6	2.864681000	-2.026905000	-0.295212000
6	1.413648000	-2.043143000	-0.504963000
6	1.409111000	0.422045000	-0.104514000
1	3.341188000	-2.990366000	-0.394613000
6	-0.673379000	-0.803120000	0.116097000
6	-3.554062000	-0.885589000	0.128763000
6	-1.409111000	0.422046000	0.104514000
6	-1.413648000	-2.043143000	0.504963000
6	-2.864681000	-2.026905000	0.295212000
6	-2.833762000	0.396379000	0.113119000
1	-3.341188000	-2.990366000	0.394613000
6	-5.026986000	-0.874019000	0.003742000
6	-7.825831000	-0.816332000	-0.202019000
6	-5.763097000	-2.056911000	-0.111376000
6	-5.725544000	0.337134000	-0.003872000
6	-7.115222000	0.361472000	-0.100709000
6	-7.140316000	-2.028095000	-0.212585000
1	-5.261293000	-3.012300000	-0.133810000
1	-7.607647000	1.323723000	-0.098348000
1	-7.685945000	-2.957330000	-0.303435000
1	-8.903866000	-0.799798000	-0.279549000
6	3.524014000	1.592533000	-0.074113000
6	2.836300000	2.816371000	-0.029689000
1	3.421229000	3.724691000	-0.008279000
6	1.472876000	2.845626000	0.005243000
1	0.973952000	3.799895000	0.064206000
6	0.722400000	1.649778000	-0.021670000
6	-0.722400000	1.649778000	0.021670000
6	-1.472876000	2.845626000	-0.005243000
1	-0.973952000	3.799895000	-0.064206000
6	-2.836300000	2.816371000	0.029689000
1	-3.421229000	3.724691000	0.008279000
6	-3.524014000	1.592533000	0.074113000
6	-5.010124000	1.632987000	0.068922000
8	-5.603257000	2.688785000	0.103853000
6	5.010124000	1.632987000	-0.068923000
8	5.603257000	2.688785000	-0.103853000
8	0.876714000	-2.965008000	-1.073220000
8	-0.876714000	-2.965008000	1.073220000

E(M06-2X): -1604.9419331

Violanthrone-16,17-quinone 10, lowest triplet excited state, M06-2X/cc-pVTZ

6	5.749163000	0.348839000	0.063165000
6	7.175531000	-2.012141000	-0.126983000
6	5.052653000	-0.854024000	-0.106479000
6	7.141461000	0.365644000	0.127388000
6	7.857995000	-0.808572000	0.030344000
6	5.796054000	-2.034950000	-0.193067000
1	7.631321000	1.320562000	0.256747000
1	8.937636000	-0.796858000	0.081122000
1	5.297149000	-2.985935000	-0.302803000
1	7.725781000	-2.940690000	-0.194935000
6	3.578324000	-0.860110000	-0.180024000
6	0.719137000	-0.811084000	-0.109828000
6	2.862601000	0.377063000	-0.001533000
6	2.865360000	-1.980867000	-0.464209000
6	1.418670000	-2.004483000	-0.531237000
6	1.434485000	0.389111000	-0.000688000
1	3.335601000	-2.922024000	-0.707717000
6	-0.719137000	-0.811084000	0.109828000
6	-3.578324000	-0.860110000	0.180024000
6	-1.434485000	0.389111000	0.000688000
6	-1.418670000	-2.004483000	0.531237000
6	-2.865360000	-1.980867000	0.464209000
6	-2.862601000	0.377063000	0.001533000
1	-3.335601000	-2.922024000	0.707717000
6	-5.052653000	-0.854024000	0.106479000
6	-7.857995000	-0.808572000	-0.030344000
6	-5.796054000	-2.034950000	0.193067000
6	-5.749163000	0.348839000	-0.063165000
6	-7.141461000	0.365644000	-0.127389000
6	-7.175531000	-2.012141000	0.126983000
1	-5.297149000	-2.985935000	0.302803000
1	-7.631321000	1.320562000	-0.256747000
1	-7.725781000	-2.940690000	0.194935000
1	-8.937636000	-0.796858000	-0.081122000
6	3.547891000	1.591275000	0.158779000
6	2.847458000	2.774419000	0.287030000
1	3.411557000	3.687954000	0.415734000
6	1.454695000	2.796061000	0.229043000
1	0.950832000	3.747003000	0.311172000
6	0.732072000	1.629984000	0.070790000
6	-0.732072000	1.629984000	-0.070789000
6	-1.454695000	2.796061000	-0.229043000
1	-0.950832000	3.747003000	-0.311173000
6	-2.847458000	2.774419000	-0.287030000
1	-3.411557000	3.687954000	-0.415734000
6	-3.547891000	1.591275000	-0.158779000
6	-5.033246000	1.639632000	-0.189770000
8	-5.621214000	2.691626000	-0.321917000
6	5.033246000	1.639632000	0.189770000
8	5.621214000	2.691626000	0.321917000
8	0.828616000	-2.989725000	-1.001477000
8	-0.828616000	-2.989725000	1.001478000

E(M06-2X): -1604.8812086

Transition state for conversion of 10 into oxirane 14, M06-2X/cc-pVTZ

6	5.583823000	0.118006000	-0.417604000
6	6.748357000	-2.388347000	-0.630716000
6	4.781930000	-1.025774000	-0.256910000
6	6.941890000	-0.004201000	-0.690509000
6	7.527927000	-1.250732000	-0.801441000
6	5.396829000	-2.276359000	-0.361300000
1	7.517743000	0.902925000	-0.809076000
1	8.584175000	-1.339913000	-1.012933000
1	4.824503000	-3.180172000	-0.219092000
1	7.196719000	-3.369515000	-0.703638000
6	3.339321000	-0.892008000	0.028548000
6	0.722089000	-0.514059000	0.693883000
6	2.829600000	0.459218000	0.208188000
6	2.502947000	-1.995955000	0.072260000
6	1.137189000	-1.813881000	0.402497000
6	1.472830000	0.636348000	0.506987000
1	2.863475000	-2.987109000	-0.150903000
6	-0.627336000	-0.510047000	0.847677000
6	-3.272201000	-0.842923000	0.062528000
6	-1.363999000	0.633957000	0.640562000
6	-1.154087000	-1.892884000	0.865823000
6	-2.460218000	-1.966360000	0.149878000
6	-2.736397000	0.466604000	0.297532000
1	-2.785585000	-2.957121000	-0.116377000
6	-4.688511000	-0.939471000	-0.301803000
6	-7.411978000	-1.089821000	-1.018084000
6	-5.333275000	-2.175692000	-0.459524000
6	-5.458986000	0.223201000	-0.490180000
6	-6.806799000	0.134569000	-0.847171000
6	-6.663007000	-2.248985000	-0.815391000
1	-4.795016000	-3.094077000	-0.278334000
1	-7.351390000	1.059321000	-0.978559000
1	-7.129094000	-3.218972000	-0.926187000
1	-8.455014000	-1.154176000	-1.294069000
6	3.592306000	1.613876000	0.025171000
6	2.983858000	2.864016000	0.115634000
1	3.605336000	3.736334000	-0.032780000~
6	1.617717000	3.004402000	0.336768000
1	1.189251000	3.997163000	0.337274000
6	0.805911000	1.881468000	0.519504000
6	-0.669118000	1.883007000	0.547356000
6	-1.454421000	3.013360000	0.341411000
1	-1.007706000	3.995203000	0.279603000
6	-2.824928000	2.878679000	0.142829000
1	-3.434690000	3.753955000	-0.041255000
6	-3.465510000	1.643073000	0.076027000
6	-4.892157000	1.579159000	-0.309998000
8	-5.548686000	2.589757000	-0.475038000

6	5.035034000	1.500231000	-0.297827000
8	5.730349000	2.478320000	-0.452871000
8	0.152540000	-2.678063000	0.403068000
8	-1.739513000	-2.437252000	1.881179000

E(M06-2X): -1604.8568706

Oxirane 14, M06-2X/cc-pVTZ

6	5.600496000	0.114645000	-0.421909000
6	6.767266000	-2.393971000	-0.596878000
6	4.798757000	-1.027212000	-0.234748000
6	6.959539000	-0.013537000	-0.698694000
6	7.547479000	-1.258716000	-0.790515000
6	5.418378000	-2.278625000	-0.322210000
1	7.532271000	0.892990000	-0.836208000
1	8.602781000	-1.351075000	-1.005272000
1	4.847797000	-3.181197000	-0.163970000
1	7.215465000	-3.376407000	-0.656393000
6	3.359753000	-0.888964000	0.053608000
6	0.749359000	-0.498354000	0.681565000
6	2.851340000	0.452808000	0.183989000
6	2.515643000	-1.999186000	0.154046000
6	1.172341000	-1.784951000	0.481110000
6	1.493348000	0.643816000	0.474996000
1	2.882034000	-2.998094000	-0.021899000
6	-0.619728000	-0.493512000	0.852961000
6	-3.302847000	-0.822741000	0.175758000
6	-1.368739000	0.620829000	0.671841000
6	-1.047932000	-1.867638000	0.812048000
6	-2.491305000	-2.038509000	0.345304000
6	-2.766832000	0.444858000	0.391289000
1	-2.735001000	-2.926263000	-0.219168000
6	-4.679148000	-0.931168000	-0.292996000
6	-7.356222000	-1.110038000	-1.160778000
6	-5.288720000	-2.173826000	-0.529175000
6	-5.467474000	0.224371000	-0.477652000
6	-6.789310000	0.121735000	-0.903492000
6	-6.595669000	-2.259785000	-0.965984000
1	-4.746267000	-3.089448000	-0.348116000
1	-7.349887000	1.038713000	-1.022408000
1	-7.032380000	-3.233452000	-1.142975000
1	-8.381063000	-1.182890000	-1.496354000
6	3.613887000	1.615901000	-0.021445000
6	2.996118000	2.855484000	0.040042000
1	3.612994000	3.728683000	-0.125203000
6	1.620632000	3.003023000	0.258698000
1	1.196082000	3.997351000	0.237960000
6	0.815477000	1.889675000	0.465254000
6	-0.672026000	1.884506000	0.525565000
6	-1.457576000	2.990021000	0.314625000
1	-1.016343000	3.969696000	0.196271000
6	-2.859727000	2.858016000	0.162027000

1	-3.459555000	3.737141000	-0.036079000
6	-3.504323000	1.648503000	0.156508000
6	-4.934266000	1.582946000	-0.213174000
8	-5.618677000	2.581564000	-0.325219000
6	5.056024000	1.499684000	-0.331600000
8	5.755111000	2.475538000	-0.502361000
8	0.111700000	-2.659207000	0.562815000
8	-1.994745000	-2.358679000	1.664755000

E(M06-2X): -1604.8878177

Transition state for conversion of 10 into peroxide 15, M06-2X/cc-pVTZ

6	-3.465412000	-0.849857000	-0.019279000
6	-2.825229000	0.417810000	0.198488000
6	-1.366448000	-1.970256000	0.468779000
6	-1.430025000	0.463238000	0.421373000
6	-2.710451000	-1.991114000	0.009681000
6	-0.707659000	-0.741500000	0.619843000
6	-0.741922000	1.705426000	0.420260000
1	-3.131705000	-2.963941000	-0.188239000
6	0.726864000	1.714980000	0.377197000
6	1.421917000	0.478094000	0.372674000
6	2.820962000	0.419897000	0.155694000
6	0.710713000	-0.717318000	0.619896000
6	3.481053000	-0.851467000	0.041898000
6	2.747096000	-1.997238000	0.211485000
1	3.186378000	-2.981041000	0.151737000
6	1.399612000	-1.930624000	0.637502000
8	-0.826068000	-3.135823000	0.697680000
8	0.820953000	-3.009103000	1.098042000
6	-1.493780000	2.872867000	0.356213000
1	-1.006725000	3.835966000	0.373986000
6	-2.874812000	2.832097000	0.234191000
1	-3.455959000	3.742021000	0.179432000
6	-3.538805000	1.624728000	0.121527000
6	1.471536000	2.882412000	0.260859000
1	0.986912000	3.846649000	0.271201000
6	2.849047000	2.834886000	0.100208000
1	3.426215000	3.744295000	0.003246000
6	3.523778000	1.629456000	0.024427000
6	4.986756000	1.638608000	-0.208472000
8	5.609281000	2.677712000	-0.284194000
6	5.647105000	0.318176000	-0.356973000
6	6.981773000	-2.085469000	-0.668978000
6	4.926616000	-0.879179000	-0.242497000
6	7.016043000	0.304471000	-0.620588000
6	7.687228000	-0.889535000	-0.777419000
6	5.626680000	-2.080135000	-0.404830000
1	7.524259000	1.255497000	-0.697548000
1	8.748441000	-0.898910000	-0.982671000
1	5.113378000	-3.026576000	-0.326244000

1	7.495523000	-3.029408000	-0.790671000
6	-5.003077000	1.628926000	-0.110693000
8	-5.637914000	2.661941000	-0.127587000
6	-5.650603000	0.310743000	-0.330913000
6	-6.961573000	-2.088588000	-0.754494000
6	-4.915594000	-0.882052000	-0.289856000
6	-7.020482000	0.295710000	-0.582905000
6	-7.679568000	-0.897163000	-0.797319000
6	-5.603432000	-2.081200000	-0.501812000
1	-7.540605000	1.243122000	-0.604113000
1	-8.742527000	-0.907738000	-0.993333000
1	-5.082233000	-3.025580000	-0.464079000
1	-7.466819000	-3.031156000	-0.915225000

E(M06-2X): -1604.8639242

Peroxide 15, M06-2X/cc-pVTZ

6	0.004162000	5.719028000	0.311477000
6	0.055079000	7.076162000	-2.100210000
6	0.038738000	4.983061000	-0.882716000
6	0.000243000	7.112098000	0.289357000
6	0.026923000	7.795027000	-0.908783000
6	0.060030000	5.695024000	-2.086805000
1	-0.025523000	7.631380000	1.237340000
1	0.023340000	8.875864000	-0.923457000
1	0.074932000	5.171847000	-3.030840000
1	0.071381000	7.598479000	-3.047216000
6	0.045754000	3.509686000	-0.847724000
6	-0.002291000	0.713773000	-0.722577000
6	-0.020078000	2.852360000	0.417160000
6	0.126889000	2.750552000	-2.002700000
6	0.107251000	1.360473000	-1.924103000
6	-0.036770000	1.439481000	0.477455000
1	0.217244000	3.200607000	-2.979444000
6	0.002291000	-0.713773000	-0.722577000
6	-0.045754000	-3.509686000	-0.847724000
6	0.036770000	-1.439481000	0.477455000
6	-0.107251000	-1.360473000	-1.924103000
6	-0.126889000	-2.750552000	-2.002700000
6	0.020078000	-2.852360000	0.417160000
1	-0.217244000	-3.200607000	-2.979444000
6	-0.038738000	-4.983061000	-0.882716000
6	-0.026923000	-7.795027000	-0.908783000
6	-0.060030000	-5.695024000	-2.086805000
6	-0.004162000	-5.719028000	0.311477000
6	-0.000243000	-7.112098000	0.289357000
6	-0.055079000	-7.076162000	-2.100210000
1	-0.074932000	-5.171847000	-3.030840000
1	0.025523000	-7.631380000	1.237340000
1	-0.071381000	-7.598479000	-3.047216000
1	-0.023340000	-8.875864000	-0.923457000
6	-0.042263000	3.569469000	1.634044000

6	-0.055079000	2.889919000	2.831106000
1	-0.065523000	3.470056000	3.743857000
6	-0.036073000	1.493526000	2.877708000
1	-0.022298000	1.012450000	3.844529000
6	-0.024584000	0.739858000	1.718578000
6	0.024584000	-0.739858000	1.718578000
6	0.036073000	-1.493526000	2.877708000
1	0.022298000	-1.012450000	3.844529000
6	0.055079000	-2.889919000	2.831106000
1	0.065523000	-3.470056000	3.743857000
6	0.042263000	-3.569469000	1.634044000
6	0.032040000	-5.051224000	1.637888000
8	0.053327000	-5.684988000	2.672355000
6	-0.032040000	5.051224000	1.637888000
8	-0.053327000	5.684988000	2.672355000
8	0.351561000	0.625315000	-3.067315000
8	-0.351561000	-0.625315000	-3.067315000

E(M06-2X): -1604.8857033

Peroxide 15, lowest triplet excited state, UM06-2X/cc-pVTZ
(n.b: in exact C_{2v} symmetry, this species is a TS at this level of theory)

6	-0.000015000	5.754844000	0.398336000
6	0.000016000	7.219241000	-1.945166000
6	0.000000000	5.075568000	-0.824162000
6	-0.000017000	7.147122000	0.445136000
6	-0.000001000	7.882861000	-0.721353000
6	0.000017000	5.839090000	-1.996520000
1	-0.000030000	7.621596000	1.416573000
1	-0.000002000	8.963314000	-0.687253000
1	0.000031000	5.359833000	-2.963488000
1	0.000029000	7.784844000	-2.867003000
6	-0.000001000	3.599641000	-0.851852000
6	0.000000000	0.708853000	-0.869407000
6	-0.000009000	2.857303000	0.386054000
6	0.000003000	2.909974000	-2.017058000
6	0.000001000	1.478230000	-2.078025000
6	-0.000004000	1.432743000	0.366274000
1	0.000006000	3.398261000	-2.978713000
6	0.000000000	-0.708853000	-0.869407000
6	0.000001000	-3.599641000	-0.851852000
6	0.000004000	-1.432743000	0.366274000
6	-0.000001000	-1.478230000	-2.078025000
6	-0.000003000	-2.909974000	-2.017058000
6	0.000009000	-2.857303000	0.386054000
1	-0.000006000	-3.398261000	-2.978713000
6	0.000000000	-5.075568000	-0.824162000
6	0.000001000	-7.882861000	-0.721353000
6	-0.000017000	-5.839090000	-1.996520000
6	0.000015000	-5.754844000	0.398336000
6	0.000017000	-7.147122000	0.445136000
6	-0.000016000	-7.219241000	-1.945166000

1	-0.000031000	-5.359833000	-2.963488000
1	0.000030000	-7.621596000	1.416573000
1	-0.000029000	-7.784844000	-2.867003000
1	0.000002000	-8.963314000	-0.687253000
6	-0.000021000	3.531648000	1.608751000
6	-0.000028000	2.824765000	2.802518000
1	-0.000040000	3.381756000	3.728622000
6	-0.000020000	1.450290000	2.792891000
1	-0.000027000	0.936022000	3.739547000
6	-0.000006000	0.728872000	1.594600000
6	0.000006000	-0.728872000	1.594600000
6	0.000020000	-1.450290000	2.792891000
1	0.000027000	-0.936022000	3.739547000
6	0.000028000	-2.824765000	2.802518000
1	0.000040000	-3.381756000	3.728622000
6	0.000021000	-3.531648000	1.608751000
6	0.000029000	-5.014502000	1.681186000
8	0.000047000	-5.589580000	2.748880000
6	-0.000029000	5.014502000	1.681186000
8	-0.000047000	5.589580000	2.748880000
8	0.000000000	0.995082000	-3.251569000
8	0.000000000	-0.995082000	-3.251569000

E(M06-2X): -1604.8897545

Phenanthrene-2,7-quinone 16, M06-2X/cc-pVTZ

6	0.000000000	0.684507000	-0.333781000
6	0.000000000	-0.684507000	-0.333781000
6	0.000000000	1.418384000	0.934350000
6	0.000000000	-1.418384000	0.934350000
6	0.000000000	0.667026000	2.176812000
1	0.000000000	1.229280000	3.101121000
6	0.000000000	-0.667026000	2.176812000
1	0.000000000	-1.229280000	3.101121000
6	0.000000000	-2.767374000	0.947742000
1	0.000000000	-3.318483000	1.879564000
6	0.000000000	2.767374000	0.947742000
1	0.000000000	3.318483000	1.879564000
6	0.000000000	-1.474982000	-1.557570000
1	0.000000000	-0.964466000	-2.507983000
6	0.000000000	-2.811670000	-1.545043000
1	0.000000000	-3.395059000	-2.455411000
6	0.000000000	-3.568192000	-0.281586000
6	0.000000000	1.474982000	-1.557570000
1	0.000000000	0.964466000	-2.507983000
6	0.000000000	2.811670000	-1.545043000
1	0.000000000	3.395059000	-2.455411000
6	0.000000000	3.568192000	-0.281586000
8	0.000000000	-4.783536000	-0.257854000
8	0.000000000	4.783536000	-0.257854000

E(M06-2X): -688.6747128

Phenanthrene-2,7-quinone 16, lowest triplet excited state, M06-2X/cc-pVTZ

6	0.000000000	0.718105000	-0.322736000
6	0.000000000	-0.718105000	-0.322736000
6	0.000000000	1.400414000	0.911518000
6	0.000000000	-1.400414000	0.911518000
6	0.000000000	0.677747000	2.137771000
1	0.000000000	1.233676000	3.065877000
6	0.000000000	-0.677747000	2.137771000
1	0.000000000	-1.233676000	3.065877000
6	0.000000000	-2.812836000	0.927303000
1	0.000000000	-3.352279000	1.865289000
6	0.000000000	2.812836000	0.927303000
1	0.000000000	3.352279000	1.865289000
6	0.000000000	-1.492924000	-1.533372000
1	0.000000000	-0.982487000	-2.484404000
6	0.000000000	-2.842223000	-1.524386000
1	0.000000000	-3.419361000	-2.438481000
6	0.000000000	-3.585136000	-0.275970000
6	0.000000000	1.492924000	-1.533372000
1	0.000000000	0.982487000	-2.484404000
6	0.000000000	2.842223000	-1.524386000
1	0.000000000	3.419361000	-2.438481000
6	0.000000000	3.585136000	-0.275970000
8	0.000000000	-4.830431000	-0.241131000
8	0.000000000	4.830431000	-0.241131000

E(M06-2X): -688.6434166

Phenanthrene-2,5-quinone 17, M06-2X/cc-pVTZ

6	-0.517319000	2.307458000	0.084906000
1	-1.111972000	3.210301000	0.125012000
6	0.816775000	2.353066000	0.073983000
1	1.345813000	3.296791000	0.098688000
6	1.605713000	1.141512000	0.021445000
6	-1.220445000	1.042383000	0.026902000
6	-0.441407000	-0.205507000	-0.014967000
6	0.927773000	-0.149819000	0.034733000
6	-2.569621000	1.010331000	0.031098000
1	-3.150457000	1.922441000	0.084195000
6	2.956809000	1.214933000	-0.072843000
1	3.430251000	2.187632000	-0.097106000
6	1.796478000	-1.379867000	0.090933000
8	1.369378000	-2.492972000	0.334005000
6	3.237504000	-1.187667000	-0.114356000
1	3.827548000	-2.092032000	-0.154771000
6	3.778124000	0.038050000	-0.167329000
1	4.848286000	0.159931000	-0.272335000
6	-1.192686000	-1.453303000	-0.129911000
1	-0.633291000	-2.368873000	-0.209873000
6	-2.529474000	-1.474289000	-0.137626000
1	-3.083580000	-2.398111000	-0.233776000

6	-3.327822000	-0.242191000	-0.034020000
8	-4.543755000	-0.258105000	-0.021219000

E(M06-2X): -688.6695285

Phenanthrene-2,5-quinone 17, lowest triplet excited state, M06-2X/cc-pVTZ

6	-2.271877000	-0.438708000	0.000000000
1	-3.341376000	-0.273221000	0.000000000
6	-1.761533000	-1.697762000	0.000000000
1	-2.414494000	-2.560321000	0.000000000
6	-0.360941000	-1.901295000	0.000000000
6	-1.410745000	0.687153000	0.000000000
6	0.000000000	0.516150000	0.000000000
6	0.525661000	-0.811007000	0.000000000
6	-1.967395000	1.977974000	0.000000000
1	-3.041148000	2.113063000	0.000000000
6	0.118968000	-3.255352000	0.000000000
1	-0.615067000	-4.049987000	0.000000000
6	1.975444000	-1.113351000	0.000000000
8	2.861613000	-0.253746000	0.000000000
6	2.372658000	-2.505690000	0.000000000
1	3.439836000	-2.677879000	0.000000000
6	1.462451000	-3.545412000	0.000000000
1	1.801280000	-4.571921000	0.000000000
6	0.819901000	1.705307000	0.000000000
1	1.888231000	1.580248000	0.000000000
6	0.283495000	2.943252000	0.000000000
1	0.908098000	3.826063000	0.000000000
6	-1.153234000	3.159055000	0.000000000
8	-1.664421000	4.290256000	0.000000000

E(M06-2X): -688.6532225

Phenanthrene-1,4,5,8-quinone 18, M06-2X/cc-pVTZ

6	-0.019627000	-1.380715000	-0.943489000
6	0.019627000	1.380715000	-0.943489000
6	-0.029193000	-0.688305000	-2.152497000
6	0.029193000	-0.699546000	0.271151000
6	-0.029193000	0.699546000	0.271151000
6	0.029193000	0.688305000	-2.152497000
1	-0.064905000	-1.257174000	-3.070999000
1	0.064905000	1.257174000	-3.070999000
6	0.104104000	2.876111000	-0.979369000
6	-0.311512000	1.496760000	1.510049000
6	0.006735000	2.946902000	1.467455000
1	-0.007260000	3.452289000	2.424085000
6	0.171661000	3.589743000	0.315196000
1	0.323834000	4.659106000	0.254168000
8	0.145338000	3.477244000	-2.026436000

8	-0.889466000	1.032488000	2.459157000
6	-0.104104000	-2.876111000	-0.979369000
6	0.311512000	-1.496760000	1.510049000
6	-0.006735000	-2.946902000	1.467455000
1	0.007260000	-3.452289000	2.424085000
6	-0.171661000	-3.589743000	0.315196000
1	-0.323834000	-4.659106000	0.254168000
8	-0.145338000	-3.477244000	-2.026436000
8	0.889466000	-1.032488000	2.459157000

E(M06-2X): -837.9390946

Phenanthrene-1,4,5,8-quinone 18, lowest triplet excited state, M06-2X/cc-pVTZ

6	0.164929000	-1.386232000	-0.968276000
6	-0.164929000	1.386232000	-0.968276000
6	0.084541000	-0.663281000	-2.227874000
6	0.155667000	-0.658783000	0.296284000
6	-0.155667000	0.658783000	0.296284000
6	-0.084541000	0.663281000	-2.227874000
1	0.143394000	-1.246268000	-3.135114000
1	-0.143394000	1.246268000	-3.135114000
6	-0.119749000	2.832245000	-0.975757000
6	-0.513222000	1.424826000	1.537754000
6	-0.259773000	2.878614000	1.490088000
1	-0.300099000	3.394269000	2.440098000
6	-0.108173000	3.522230000	0.330601000
1	-0.000216000	4.597976000	0.281590000
8	-0.084541000	3.460102000	-2.032075000
8	-1.069366000	0.904604000	2.471639000
6	0.119749000	-2.832245000	-0.975757000
6	0.513222000	-1.424826000	1.537754000
6	0.259773000	-2.878614000	1.490088000
1	0.300099000	-3.394269000	2.440098000
6	0.108173000	-3.522230000	0.330601000
1	0.000216000	-4.597976000	0.281590000
8	0.084541000	-3.460102000	-2.032075000
8	1.069366000	-0.904604000	2.471639000

E(M06-2X): -837.8280779

Peroxide 21, lowest triplet state, M06-2X/cc-pVTZ (n.b: this species is not a minimum on the singlet PES)

6	0.000000000	1.395944000	-0.953456000
6	0.000000000	0.679869000	-2.172167000
1	0.000000000	1.253931000	-3.087603000
6	0.000000000	-0.679869000	-2.172167000
1	0.000000000	-1.253931000	-3.087603000
6	0.000000000	-1.395944000	-0.953456000
6	0.000000000	-2.861391000	-1.017316000

6	0.000000000	2.861391000	-1.017316000
6	0.000000000	0.705612000	0.263713000
6	0.000000000	-0.705612000	0.263713000
6	0.000000000	-1.495067000	1.506895000
6	0.000000000	1.495067000	1.506895000
6	0.000000000	2.940269000	1.438880000
1	0.000000000	3.460634000	2.385768000
6	0.000000000	3.578874000	0.257980000
1	0.000000000	4.659204000	0.197276000
6	0.000000000	-2.940269000	1.438880000
1	0.000000000	-3.460634000	2.385768000
6	0.000000000	-3.578874000	0.257980000
1	0.000000000	-4.659204000	0.197276000
8	0.000000000	-3.468184000	-2.079902000
8	0.000000000	3.468184000	-2.079902000
8	0.000000000	0.995175000	2.649574000
8	0.000000000	-0.995175000	2.649574000

E(M06-2X)/cc-pVTZ: -837.8426013

Phenanthrene-4,5,9,10-quinone 19, M06-2X/cc-pVTZ

1	-0.430942000	4.560681000	-0.485158000
6	-0.227347000	3.498537000	-0.505171000
1	-0.281563000	3.326831000	1.684902000
6	-0.162031000	2.787285000	0.754437000
6	0.381522000	1.446328000	-1.673128000
6	0.001310000	1.448393000	0.809002000
6	-0.012579000	2.867497000	-1.667813000
6	0.077778000	0.673980000	-0.417163000
6	-0.012579000	0.772566000	2.132856000
1	-0.002827000	3.376521000	-2.621203000
6	0.012579000	-0.772566000	2.132856000
6	-0.001310000	-1.448393000	0.809002000
6	0.162031000	-2.787285000	0.754437000
6	-0.077778000	-0.673980000	-0.417163000
6	0.227347000	-3.498537000	-0.505171000
1	0.281563000	-3.326831000	1.684902000
1	0.430942000	-4.560681000	-0.485158000
6	0.012579000	-2.867497000	-1.667813000
1	0.002827000	-3.376521000	-2.621203000
6	-0.381522000	-1.446328000	-1.673128000
8	-0.036076000	1.365890000	3.175467000
8	0.036076000	-1.365890000	3.175467000
8	-1.031997000	-0.971912000	-2.572551000
8	1.031997000	0.971912000	-2.572551000

E(M06-2X): -837.8893028

Phenanthrene-4,5,9,10-quinone 19, lowest triplet excited state, UM06-2X/cc-pVTZ

1	-0.158723000	4.649997000	-0.414787000
6	-0.076966000	3.572246000	-0.426590000
1	-0.432606000	3.345583000	1.697219000
6	-0.246987000	2.850023000	0.754346000
6	0.409639000	1.474119000	-1.616644000
6	-0.172812000	1.446536000	0.754122000
6	0.246987000	2.910505000	-1.582427000
6	0.031087000	0.733118000	-0.411427000
6	-0.227833000	0.738641000	2.059773000
1	0.461766000	3.432471000	-2.504599000
6	0.227833000	-0.738641000	2.059773000
6	0.172812000	-1.446536000	0.754122000
6	0.246987000	-2.850023000	0.754346000
6	-0.031087000	-0.733118000	-0.411427000
6	0.076966000	-3.572246000	-0.426590000
1	0.432606000	-3.345583000	1.697219000
1	0.158723000	-4.649997000	-0.414787000
6	-0.246987000	-2.910505000	-1.582427000
1	-0.461766000	-3.432471000	-2.504599000
6	-0.409639000	-1.474119000	-1.616644000
8	-0.542168000	1.268422000	3.089143000
8	0.542168000	-1.268422000	3.089143000
8	-0.948539000	-0.923546000	-2.584737000
8	0.948539000	0.923546000	-2.584737000

E(M06-2X): -837.869352

Peroxide 22, M06-2X/cc-pVTZ

6	0.776179000	2.126696000	0.095632000
6	-0.776179000	2.126696000	-0.095631000
8	-1.344228000	3.176791000	-0.212185000
8	1.344229000	3.176791000	0.212186000
6	-1.481698000	0.818424000	-0.088824000
6	-2.871204000	0.743450000	-0.088509000
1	-3.443558000	1.658178000	-0.150939000
6	1.481698000	0.818424000	0.088825000
6	2.871205000	0.743449000	0.088508000
1	3.443559000	1.658177000	0.150937000
6	3.489266000	-0.492729000	-0.009629000
1	4.568143000	-0.556910000	-0.011155000
6	2.739476000	-1.663031000	-0.116755000
1	3.209699000	-2.630962000	-0.215790000
6	1.361956000	-1.571436000	-0.111556000
6	0.724941000	-0.344153000	0.006750000
6	-0.724941000	-0.344152000	-0.006748000
6	-1.361956000	-1.571435000	0.111558000
6	-2.739477000	-1.663031000	0.116754000
1	-3.209700000	-2.630961000	0.215788000
6	-3.489266000	-0.492728000	0.009624000
1	-4.568144000	-0.556909000	0.011148000

8	0.612995000	-2.698496000	-0.370090000
8	-0.612996000	-2.698495000	0.370092000

E(M06-2X): -837.8794716

Peroxide 22, lowest triplet excited state, M06-2X/cc-pVTZ

6	-0.752852000	2.159889000	-0.039134000
6	0.752850000	2.159892000	0.039128000
8	1.386117000	3.204296000	0.085419000
8	-1.386124000	3.204294000	-0.085405000
6	1.433032000	0.858565000	0.038544000
6	2.815403000	0.767657000	0.034638000
1	3.385807000	1.685952000	0.052999000
6	-1.433033000	0.858565000	-0.038546000
6	-2.815406000	0.767651000	-0.034646000
1	-3.385813000	1.685944000	-0.053011000
6	-3.451355000	-0.471145000	-0.002811000
1	-4.531902000	-0.508931000	-0.008989000
6	-2.739944000	-1.683780000	0.036367000
1	-3.239347000	-2.639686000	0.077552000
6	-1.374703000	-1.614981000	0.038391000
6	-0.685240000	-0.364338000	-0.012947000
6	0.685238000	-0.364337000	0.012952000
6	1.374704000	-1.614976000	-0.038388000
6	2.739949000	-1.683772000	-0.036377000
1	3.239346000	-2.639681000	-0.077565000
6	3.451358000	-0.471141000	0.002796000
1	4.531906000	-0.508920000	0.008967000
8	-0.676079000	-2.760369000	0.223007000
8	0.676086000	-2.760368000	-0.222990000

E(M06-2X): -837.7930881

Quinone 20, lowest open-shell singlet state, UM06-2X/cc-pVTZ (broken-symmetry wavefunction)

1	0.033191000	5.301750000	-3.207834000
6	-0.044639000	5.784370000	-2.245670000
6	-0.266068000	7.097071000	0.196571000
6	-0.054788000	5.009216000	-1.071316000
6	-0.140370000	7.152414000	-2.197793000
6	-0.244813000	7.818202000	-0.965081000
6	-0.182578000	5.686011000	0.165525000
1	-0.131378000	7.721649000	-3.116971000
1	-0.305503000	8.897288000	-0.937707000
1	-0.329227000	7.589566000	1.156333000
6	0.054788000	3.561438000	-1.092433000
6	0.091412000	0.685912000	-1.053372000
6	0.273910000	2.862941000	-2.259898000
6	-0.000054000	2.842718000	0.147325000
6	0.038582000	1.409509000	0.164945000
6	0.463052000	1.431727000	-2.288709000

1	0.434109000	3.354929000	-3.206741000
6	-0.091412000	-0.685912000	-1.053372000
6	-0.054788000	-3.561438000	-1.092433000
6	-0.463052000	-1.431727000	-2.288709000
6	-0.038582000	-1.409509000	0.164945000
6	0.000054000	-2.842718000	0.147325000
6	-0.273910000	-2.862941000	-2.259898000
1	-0.434109000	-3.354929000	-3.206741000
6	0.054788000	-5.009216000	-1.071316000
6	0.244813000	-7.818202000	-0.965081000
6	0.044639000	-5.784370000	-2.245670000
6	0.182578000	-5.686011000	0.165525000
6	0.266068000	-7.097071000	0.196571000
6	0.140370000	-7.152414000	-2.197793000
1	-0.033191000	-5.301750000	-3.207834000
1	0.329227000	-7.589566000	1.156333000
1	0.131378000	-7.721649000	-3.116971000
1	0.305503000	-8.897288000	-0.937707000
6	-0.225945000	4.934753000	1.365179000
6	-0.127604000	3.543122000	1.367953000
6	-0.022289000	0.709345000	1.393095000
6	0.022289000	-0.709345000	1.393095000
6	0.127604000	-3.543122000	1.367953000
6	0.225945000	-4.934753000	1.365179000
6	-0.163576000	2.813544000	2.598201000
1	-0.224159000	3.370221000	3.522121000
6	-0.109547000	1.466035000	2.606953000
1	-0.148508000	0.954585000	3.555423000
6	0.109547000	-1.466035000	2.606953000
1	0.148508000	-0.954585000	3.555423000
6	0.163576000	-2.813544000	2.598201000
1	0.224159000	-3.370221000	3.522121000
8	-0.367420000	5.596026000	2.549181000
8	0.367420000	-5.596026000	2.549181000
8	1.014606000	0.892696000	-3.235904000
8	-1.014606000	-0.892696000	-3.235904000
6	-1.725678000	5.793234000	2.935398000
1	-1.707060000	6.338015000	3.875131000
1	-2.259071000	6.374223000	2.180058000
1	-2.227499000	4.832892000	3.072727000
6	1.725678000	-5.793234000	2.935398000
1	2.227499000	-4.832892000	3.072727000
1	1.707060000	-6.338015000	3.875131000
1	2.259071000	-6.374223000	2.180058000

E(M06-2X): -1684.6908999

Quinone 20, triplet state, UM06-2X/cc-pVTZ

1	-5.297274000	-3.203815000	-0.335387000
6	-5.781915000	-2.241191000	-0.279655000
6	-7.102299000	0.202741000	-0.120651000
6	-5.006042000	-1.067959000	-0.221389000

6	-7.152539000	-2.190736000	-0.259389000
6	-7.822339000	-0.957097000	-0.186402000
6	-5.688120000	0.169500000	-0.126395000
1	-7.721838000	-3.108880000	-0.304157000
1	-8.903085000	-0.928843000	-0.185426000
1	-7.595973000	1.163163000	-0.081096000
6	-3.555570000	-1.090354000	-0.250201000
6	-0.681046000	-1.059077000	-0.127486000
6	-2.845576000	-2.258665000	-0.432283000
6	-2.840254000	0.145860000	-0.153226000
6	-1.405377000	0.159244000	-0.113018000
6	-1.405946000	-2.292036000	-0.541318000
1	-3.329380000	-3.205196000	-0.618061000
6	0.681045000	-1.059078000	0.127469000
6	3.555569000	-1.090355000	0.250197000
6	1.405943000	-2.292042000	0.541290000
6	1.405377000	0.159243000	0.113006000
6	2.840253000	0.145859000	0.153217000
6	2.845574000	-2.258666000	0.432276000
1	3.329379000	-3.205196000	0.618060000
6	5.006041000	-1.067960000	0.221393000
6	7.822338000	-0.957098000	0.186421000
6	5.781914000	-2.241192000	0.279664000
6	5.688120000	0.169499000	0.126401000
6	7.102299000	0.202740000	0.120666000
6	7.152538000	-2.190737000	0.259406000
1	5.297272000	-3.203815000	0.335394000
1	7.595972000	1.163162000	0.081112000
1	7.721837000	-3.108880000	0.304178000
1	8.903085000	-0.928844000	0.185451000
6	-4.939300000	1.366809000	-0.037978000
6	-3.543389000	1.367009000	-0.061157000
6	-0.710367000	1.386802000	-0.014721000
6	0.710367000	1.386802000	0.014710000
6	3.543389000	1.367008000	0.061152000
6	4.939300000	1.366808000	0.037980000
6	-2.816395000	2.596196000	0.017542000
1	-3.374118000	3.520856000	0.050925000
6	-1.467855000	2.602317000	0.035261000
1	-0.957507000	3.549554000	0.104291000
6	1.467856000	2.602317000	-0.035268000
1	0.957508000	3.549554000	-0.104297000
6	2.816396000	2.596195000	-0.017547000
1	3.374119000	3.520855000	-0.050926000
8	-5.604422000	2.551448000	0.071823000
8	5.604422000	2.551447000	-0.071816000
8	-0.841023000	-3.243903000	-1.058065000
8	0.841022000	-3.243895000	1.058065000
6	-5.876466000	2.933150000	1.418599000
1	-6.418575000	3.873452000	1.372734000
1	-6.486711000	2.176527000	1.916154000
1	-4.945216000	3.067780000	1.973142000
6	5.876475000	2.933150000	-1.418591000
1	4.945228000	3.067780000	-1.973139000
1	6.418583000	3.873452000	-1.372722000

1	6.486722000	2.176527000	-1.916143000

E(M06-2X): -1684.6906163

Peroxide 23, M06-2X/cc-pVTZ

1	-5.171785000	-3.247156000	0.057274000
6	-5.681169000	-2.296314000	0.094189000
6	-7.063169000	0.113913000	0.221554000
6	-4.932775000	-1.096382000	0.098177000
6	-7.047945000	-2.284985000	0.146517000
6	-7.749736000	-1.066211000	0.214474000
6	-5.649130000	0.127654000	0.157397000
1	-7.592651000	-3.219055000	0.143815000
1	-8.829628000	-1.068414000	0.268794000
1	-7.582853000	1.058406000	0.296833000
6	-3.496664000	-1.075566000	0.051122000
6	-0.698391000	-0.968236000	0.015302000
6	-2.726025000	-2.276397000	-0.042218000
6	-2.837644000	0.167284000	0.090361000
6	-1.414448000	0.227425000	0.061741000
6	-1.373940000	-2.204080000	-0.070112000
1	-3.194330000	-3.244267000	-0.126123000
6	0.698391000	-0.968236000	-0.015303000
6	3.496664000	-1.075566000	-0.051122000
6	1.373940000	-2.204081000	0.070110000
6	1.414449000	0.227425000	-0.061741000
6	2.837644000	0.167284000	-0.090361000
6	2.726025000	-2.276397000	0.042217000
1	3.194330000	-3.244267000	0.126121000
6	4.932775000	-1.096382000	-0.098178000
6	7.749737000	-1.066211000	-0.214474000
6	5.681169000	-2.296314000	-0.094190000
6	5.649130000	0.127654000	-0.157397000
6	7.063169000	0.113914000	-0.221554000
6	7.047945000	-2.284985000	-0.146518000
1	5.171785000	-3.247156000	-0.057275000
1	7.582853000	1.058406000	-0.296832000
1	7.592651000	-3.219055000	-0.143816000
1	8.829629000	-1.068414000	-0.268794000
6	-4.942314000	1.357595000	0.160019000
6	-3.568532000	1.394212000	0.138688000
6	-0.701851000	1.464314000	0.043699000
6	0.701851000	1.464314000	-0.043699000
6	3.568532000	1.394212000	-0.138688000
6	4.942314000	1.357595000	-0.160018000
6	-2.837013000	2.634782000	0.163720000
1	-3.405242000	3.551078000	0.240563000
6	-1.490988000	2.669114000	0.113121000
1	-0.994979000	3.627372000	0.140439000
6	1.490989000	2.669114000	-0.113120000
1	0.994979000	3.627372000	-0.140437000
6	2.837013000	2.634782000	-0.163719000

1	3.405242000	3.551079000	-0.240561000
8	-5.655852000	2.526431000	0.201421000
8	5.655852000	2.526431000	-0.201420000
8	-0.625407000	-3.323321000	-0.357871000
8	0.625407000	-3.323321000	0.357870000
6	-5.999908000	3.020435000	-1.087716000
1	-6.577482000	3.927990000	-0.933203000
1	-5.100146000	3.248193000	-1.664183000
1	-6.600193000	2.289634000	-1.634772000
6	5.999907000	3.020435000	1.087717000
1	5.100145000	3.248193000	1.664184000
1	6.600191000	2.289634000	1.634774000
1	6.577482000	3.927989000	0.933205000

E(M06-2X): -1684.6768827

Peroxide 23, lowest triplet excited state, M06-2X/cc-pVTZ

1	-5.183544000	-3.249150000	0.037907000
6	-5.699695000	-2.301814000	0.081721000
6	-7.100909000	0.090866000	0.223909000
6	-4.967897000	-1.107036000	0.092895000
6	-7.076215000	-2.305325000	0.133521000
6	-7.782516000	-1.098160000	0.207320000
6	-5.689314000	0.114717000	0.162608000
1	-7.610505000	-3.245081000	0.124177000
1	-8.862551000	-1.105544000	0.258934000
1	-7.627684000	1.031007000	0.304537000
6	-3.510973000	-1.077828000	0.040143000
6	-0.715521000	-0.951021000	0.015461000
6	-2.747350000	-2.245867000	-0.063901000
6	-2.852229000	0.179537000	0.095544000
6	-1.438291000	0.245116000	0.072184000
6	-1.367106000	-2.159944000	-0.081398000
1	-3.200288000	-3.221665000	-0.142681000
6	0.715521000	-0.951021000	-0.015461000
6	3.510973000	-1.077828000	-0.040143000
6	1.367106000	-2.159944000	0.081398000
6	1.438291000	0.245115000	-0.072184000
6	2.852229000	0.179537000	-0.095544000
6	2.747350000	-2.245867000	0.063901000
1	3.200288000	-3.221665000	0.142681000
6	4.967897000	-1.107036000	-0.092895000
6	7.782516000	-1.098159000	-0.207320000
6	5.699695000	-2.301814000	-0.081721000
6	5.689314000	0.114717000	-0.162608000
6	7.100909000	0.090866000	-0.223909000
6	7.076215000	-2.305325000	-0.133521000
1	5.183544000	-3.249150000	-0.037907000
1	7.627684000	1.031007000	-0.304537000
1	7.610505000	-3.245081000	-0.124177000
1	8.862551000	-1.105544000	-0.258934000
6	-4.992390000	1.346762000	0.172669000

6	-3.586962000	1.401743000	0.154029000
6	-0.738512000	1.491208000	0.053638000
6	0.738512000	1.491208000	-0.053638000
6	3.586962000	1.401743000	-0.154029000
6	4.992390000	1.346762000	-0.172669000
6	-2.885730000	2.615450000	0.182860000
1	-3.446070000	3.535944000	0.260738000
6	-1.496056000	2.648996000	0.122224000
1	-1.011100000	3.614538000	0.130341000
6	1.496056000	2.648996000	-0.122224000
1	1.011100000	3.614538000	-0.130342000
6	2.885730000	2.615450000	-0.182860000
1	3.446070000	3.535944000	-0.260738000
8	-5.701260000	2.511794000	0.208208000
8	5.701260000	2.511794000	-0.208208000
8	-0.628870000	-3.296993000	-0.346068000
8	0.628870000	-3.296993000	0.346068000
6	-6.023820000	3.020479000	-1.083245000
1	-6.578018000	3.942248000	-0.928332000
1	-5.114013000	3.225948000	-1.651615000
1	-6.639903000	2.306079000	-1.633931000
6	6.023820000	3.020479000	1.083245000
1	5.114013000	3.225947000	1.651615000
1	6.639903000	2.306079000	1.633931000
1	6.578018000	3.942248000	0.928333000

E(M06-2X): -1684.6239593

Oviedomycin 24, M06-2X/cc-pVTZ

1	-2.525615000	-3.332141000	0.326628000
6	-3.043757000	-2.400682000	0.152520000
6	-4.359788000	0.046165000	-0.229633000
6	-2.337903000	-1.220047000	0.198907000
6	-4.412102000	-2.359890000	-0.119516000
6	-5.063130000	-1.160621000	-0.305678000
6	-2.972347000	0.017951000	0.003451000
1	-4.974075000	-3.282080000	-0.177515000
1	-6.124739000	-1.114123000	-0.502225000
6	-0.889345000	-1.254906000	0.536205000
6	-2.199649000	1.254050000	0.067352000
6	-0.069359000	-0.053181000	0.157474000
6	1.423390000	2.301108000	0.044782000
6	1.313484000	-0.116811000	0.018590000
6	-0.728380000	1.185300000	0.120496000
6	0.029668000	2.376665000	0.054150000
6	2.049280000	1.078707000	0.010675000
1	2.008698000	3.209355000	0.040948000
8	-0.421641000	-2.153223000	1.185933000
8	-2.767066000	2.356737000	0.013673000
8	-5.051073000	1.174733000	-0.388031000
1	-4.437990000	1.925973000	-0.279095000
8	-0.519974000	3.582530000	0.018020000

1	-1.494300000	3.472133000	0.022493000
6	2.046487000	-1.368488000	-0.265432000
6	3.558872000	1.065131000	0.010458000
6	3.541019000	-1.346148000	-0.167038000
6	4.262164000	-0.219547000	-0.038434000
6	5.754339000	-0.193259000	-0.003881000
1	6.137678000	0.412767000	-0.824840000
1	6.161370000	-1.197532000	-0.072284000
1	6.098784000	0.276713000	0.917596000
8	4.100298000	-2.549347000	-0.296218000
1	3.375871000	-3.174558000	-0.469027000
8	4.165338000	2.112364000	0.062305000
8	1.538698000	-2.396980000	-0.652602000

E(M06-2X): -1256.6333974

Oviedomycin, tautomer 24b, lowest triplet excited state, M06-2X/cc-pVTZ

1	2.510999000	-3.299120000	-0.601573000
6	3.046942000	-2.384247000	-0.397109000
6	4.394442000	0.023649000	0.110833000
6	2.317835000	-1.213013000	-0.252927000
6	4.424527000	-2.354844000	-0.272297000
6	5.092957000	-1.163905000	-0.020942000
6	2.974356000	0.020077000	0.002872000
1	4.994112000	-3.268638000	-0.372465000
1	6.168800000	-1.127902000	0.073482000
6	0.854113000	-1.279590000	-0.399191000
6	2.193066000	1.199095000	0.102815000
6	0.113290000	-0.070541000	-0.059469000
6	-1.428774000	2.308242000	-0.129503000
6	-1.340245000	-0.086954000	0.023999000
6	0.775906000	1.142517000	0.062324000
6	-0.001844000	2.395406000	0.060906000
6	-2.070491000	1.085090000	-0.124674000
1	-1.993052000	3.226582000	-0.208685000
8	0.289244000	-2.274304000	-0.842932000
8	2.805791000	2.379062000	0.229356000
1	4.534051000	1.903073000	0.385775000
8	0.551740000	3.499998000	0.173326000
1	2.099087000	3.083674000	0.260655000
6	-2.086585000	-1.278683000	0.468119000
6	-3.573787000	1.059299000	-0.252087000
6	-3.566309000	-1.302079000	0.241011000
6	-4.279647000	-0.215527000	-0.104006000
6	-5.762640000	-0.214660000	-0.273133000
1	-6.220923000	0.483902000	0.426992000
1	-6.170341000	-1.208219000	-0.112873000
1	-6.025072000	0.128042000	-1.274275000
8	-4.123985000	-2.485286000	0.488348000
1	-3.418375000	-3.069599000	0.813617000
8	-4.161208000	2.092086000	-0.485469000

8	-1.593811000	-2.201017000	1.077288000

E(M06-2X): -1256.5552685

Tetrangomycin 25, M06-2X/cc-pVTZ

1	-2.509876000	-3.144589000	-0.275781000
6	-3.011812000	-2.201751000	-0.116108000
6	-4.281652000	0.270341000	0.242405000
6	-2.281947000	-1.034144000	-0.178877000
6	-4.378338000	-2.133747000	0.153545000
6	-5.009003000	-0.920686000	0.328421000
6	-2.897198000	0.214968000	0.004393000
1	-4.956347000	-3.045468000	0.220968000
1	-6.069451000	-0.853965000	0.525970000
6	-0.829463000	-1.110392000	-0.506744000
6	-2.112746000	1.449947000	-0.068073000
6	0.000092000	0.102924000	-0.220305000
6	-0.632251000	1.340831000	-0.166912000
8	-0.366767000	-2.068806000	-1.073620000
8	-2.639714000	2.554699000	0.002950000
8	-4.939686000	1.420282000	0.393640000
1	-4.292410000	2.148058000	0.285484000
6	1.397637000	0.033621000	-0.171850000
6	2.155503000	1.203639000	-0.209897000
6	1.499708000	2.438015000	-0.204190000
1	2.089473000	3.345850000	-0.216637000
6	0.126272000	2.510967000	-0.151113000
1	-0.388517000	3.460201000	-0.105878000
6	2.123293000	-1.255497000	0.088194000
6	3.522733000	-1.311365000	-0.463643000
1	3.509236000	-1.221077000	-1.551997000
1	3.975041000	-2.264224000	-0.187706000
6	4.300817000	-0.136130000	0.145206000
6	3.662275000	1.175696000	-0.295672000
1	3.944431000	1.375870000	-1.333543000
1	4.080769000	1.980114000	0.310882000
8	1.679151000	-2.101484000	0.817842000
6	5.770490000	-0.189082000	-0.239951000
1	5.896793000	-0.173446000	-1.322780000
1	6.224495000	-1.102681000	0.145671000
1	6.295918000	0.662878000	0.191046000
8	4.164294000	-0.154898000	1.561353000
1	4.275769000	-1.054794000	1.884005000

E(M06-2X): -1108.5761784

Tetrangomycin, tautomer 25b, lowest triplet excited state, M06-2X/cc-pVTZ

1	-2.555744000	-3.131285000	-0.334252000
6	-3.077644000	-2.197478000	-0.181707000

6	-4.332846000	0.307928000	0.162421000
6	-2.273710000	-1.005065000	-0.140994000
6	-4.443916000	-2.143595000	-0.028899000
6	-5.076135000	-0.917844000	0.141586000
6	-2.871098000	0.229633000	0.023527000
1	-5.026616000	-3.053532000	-0.047266000
1	-6.147473000	-0.832222000	0.256325000
6	-0.829473000	-1.138541000	-0.339738000
6	-2.080707000	1.413503000	0.021162000
6	-0.028055000	0.074085000	-0.139476000
6	-0.665742000	1.330470000	-0.072697000
8	-0.356529000	-2.203371000	-0.731161000
8	-2.640431000	2.610177000	0.113584000
8	-4.908193000	1.408283000	0.292576000
1	-3.616995000	2.469524000	0.202054000
6	1.382595000	0.021696000	-0.132237000
6	2.131125000	1.183297000	-0.227213000
6	1.470206000	2.429867000	-0.212110000
1	2.062785000	3.334638000	-0.260894000
6	0.113013000	2.509393000	-0.097781000
1	-0.388019000	3.464813000	-0.042522000
6	2.139835000	-1.240972000	0.162141000
6	3.494405000	-1.321447000	-0.490032000
1	3.409280000	-1.253805000	-1.576147000
1	3.966435000	-2.266681000	-0.220705000
6	4.309447000	-0.133732000	0.041353000
6	3.633193000	1.168940000	-0.374100000
1	3.868639000	1.375029000	-1.422982000
1	4.069209000	1.978265000	0.214039000
8	1.783707000	-2.023982000	1.002723000
6	5.747499000	-0.187819000	-0.449631000
1	5.794651000	-0.187632000	-1.538988000
1	6.231407000	-1.092792000	-0.081530000
1	6.298431000	0.672921000	-0.070649000
8	4.287010000	-0.132779000	1.464681000
1	4.257544000	-1.037766000	1.790847000

E(M06-2X): -1108.5039796

1-Hydroxyanthraquinone 26, M06-2X/cc-pVTZ

1	3.380935000	0.920030000	0.000000000
6	2.415653000	1.404267000	0.000000000
6	-0.108870000	2.613651000	0.000000000
6	1.278543000	0.623773000	0.000000000
6	2.292164000	2.792983000	0.000000000
6	1.052485000	3.393414000	0.000000000
6	0.000000000	1.211155000	0.000000000
1	3.180553000	3.409918000	0.000000000
1	0.939904000	4.468347000	0.000000000
6	1.436705000	-0.860422000	0.000000000
6	-1.213846000	0.387612000	0.000000000
6	0.196200000	-1.686390000	0.000000000
6	-2.090581000	-3.262073000	0.000000000
6	0.308134000	-3.071733000	0.000000000

6	-1.065219000	-1.090746000	0.000000000
6	-2.208388000	-1.883168000	0.000000000
6	-0.832198000	-3.856417000	0.000000000
1	1.296883000	-3.508546000	0.000000000
1	-3.174551000	-1.398901000	0.000000000
1	-0.744920000	-4.934238000	0.000000000
1	-2.978789000	-3.878717000	0.000000000
8	-1.282504000	3.244878000	0.000000000
1	-1.985602000	2.560376000	0.000000000
8	-2.332389000	0.890421000	0.000000000
8	2.530005000	-1.377011000	0.000000000

E(M06-2X): -763.9860698

1-Hydroxyanthraquinone, tautomer 26b, lowest triplet excited state, M06-2X/cc-pVTZ

1	3.385428000	0.888951000	0.000000000
6	2.436683000	1.406395000	0.000000000
6	-0.101299000	2.640795000	0.000000000
6	1.253743000	0.586291000	0.000000000
6	2.357072000	2.778915000	0.000000000
6	1.113706000	3.401052000	0.000000000
6	0.000000000	1.172253000	0.000000000
1	3.258970000	3.374256000	0.000000000
1	1.006429000	4.476571000	0.000000000
6	1.419043000	-0.866453000	0.000000000
6	-1.177803000	0.370445000	0.000000000
6	0.190680000	-1.662922000	0.000000000
6	-2.126616000	-3.218022000	0.000000000
6	0.278658000	-3.059350000	0.000000000
6	-1.080105000	-1.048217000	0.000000000
6	-2.239500000	-1.851432000	0.000000000
6	-0.861291000	-3.828063000	0.000000000
1	1.264174000	-3.503567000	0.000000000
1	-3.206927000	-1.370821000	0.000000000
1	-0.786588000	-4.906762000	0.000000000
1	-3.017272000	-3.831219000	0.000000000
8	-1.213215000	3.208776000	0.000000000
1	-2.254101000	1.903727000	0.000000000
8	-2.381134000	0.921275000	0.000000000
8	2.540854000	-1.375209000	0.000000000

E(M06-2X): -763.9145887
